# A targeted metabolomics approach for sepsis-induced ARDS and its subphenotypes

**DOI:** 10.1186/s13054-023-04552-0

**Published:** 2023-07-05

**Authors:** Youjin Chang, Hyun Ju Yoo, Su Jung Kim, Kwangha Lee, Chae-Man Lim, Sang-Bum Hong, Younsuck Koh, Jin Won Huh

**Affiliations:** 1 Division of Pulmonary and Critical Care Medicine, Department of Internal Medicine, College of Medicine, Inje University Sanggye Paik Hospital, Seoul, Republic of Korea; 2 Department of Convergence Medicine, Asan Institute for Life Sciences, Asan Medical Center, University of Ulsan College of Medicine, Seoul, Republic of Korea; 3 Department of Internal Medicine, Pusan National University School of Medicine, Busan, Republic of Korea; 4 Department of Pulmonary and Critical Care Medicine, Asan Medical Center, University of Ulsan College of Medicine, 88 Olympic-ro 43-gil, Songpa-gu, Seoul, 05505 Republic of Korea

**Keywords:** Adult, Metabolomics, Biomarkers, Pathways, Respiratory distress syndrome, Sepsis

## Abstract

**Background:**

Acute respiratory distress syndrome (ARDS) is etiologically and clinically a heterogeneous disease. Its diagnostic characteristics and subtype classification, and the application of these features to treatment, have been of considerable interest. Metabolomics is becoming important for identifying ARDS biology and distinguishing its subtypes. This study aimed to identify metabolites that could distinguish sepsis-induced ARDS patients from non-ARDS controls, using a targeted metabolomics approach, and to identify whether sepsis-induced direct and sepsis-induced indirect ARDS are metabolically distinct groups, and if so, confirm their metabolites and associated pathways.

**Methods:**

This study retrospectively analyzed 54 samples of ARDS patients from a sepsis registry that was prospectively collected from March 2011 to February 2018, along with 30 non-ARDS controls. The cohort was divided into direct and indirect ARDS. Metabolite concentrations of five analyte classes (energy metabolism, free fatty acids, amino acids, phospholipids, sphingolipids) were measured using liquid chromatography–tandem mass spectrometry and gas chromatography–mass spectrometry by targeted metabolomics.

**Results:**

In total, 186 metabolites were detected. Among them, 102 metabolites could differentiate sepsis-induced ARDS patients from the non-ARDS controls, while 14 metabolites could discriminate sepsis-induced ARDS subphenotypes. Using partial least-squares discriminant analysis, we showed that sepsis-induced ARDS patients were metabolically distinct from the non-ARDS controls. The main distinguishing metabolites were lysophosphatidylethanolamine (lysoPE) plasmalogen, PE plasmalogens, and phosphatidylcholines (PCs). Sepsis-induced direct and indirect ARDS were also metabolically distinct subgroups, with differences in lysoPCs. Glycerophospholipid and sphingolipid metabolism were the most significant metabolic pathways involved in sepsis-induced ARDS biology and in sepsis-induced direct/indirect ARDS, respectively.

**Conclusion:**

Our study demonstrated a marked difference in metabolic patterns between sepsis-induced ARDS patients and non-ARDS controls, and between sepsis-induced direct and indirect ARDS subpheonotypes. The identified metabolites and pathways can provide clues relevant to the diagnosis and treatment of individuals with ARDS.

**Supplementary Information:**

The online version contains supplementary material available at 10.1186/s13054-023-04552-0.

## Introduction

The mortality associated with acute respiratory distress syndrome (ARDS) remains above 40% despite many advances in intensive care [1]. Many clinical trials have evaluated the efficacy of certain drugs in ARDS, but these have mostly failed to improve the clinical course [2]. In clinical practice, it is important to screen patients at risk of developing ARDS and to modify risk factors as much as possible. The Lung Injury Prediction Score (LIPS) is a scoring system used to screen for patients at high risk of developing ARDS [3, 4]. However, it has some limitations as a predictive scoring system, because it has a high sensitivity but a low specificity.

In addition, since ARDS is a heterogeneous disease in terms of its causes and clinical aspects, intensivists have been interested in classifying subtypes and applying different treatments to these. ARDS subphenotypes have typically been divided into direct (pulmonary) ARDS and indirect (extrapulmonary) ARDS, according to the etiology. In direct ARDS, alveolar collapse, fibrin deposition, and pulmonic edema are more common in terms of pathological findings [5, 6]. Ground glass opacities and consolidations are relatively asymmetrical in the radiological findings in direct ARDS [7]. On the other hand, indirect ARDS shows relatively bilateral ground glass opacities, rather than asymmetrical consolidations, and with compliance, it responds better to positive end-expiratory pressure than does direct ARDS [6–8]. These clinical differences result from the differences in the main pathophysiology between direct ARDS (epithelial injury) and indirect ARDS (endothelial damage and systemic inflammation). Because these subphenotypes present different clinical courses and treatment responses, they may be helpful for distinguishing the ARDS phenotype. Although biological discrimination of the two groups is not easy due to overlapping etiologies related to lung damage, several recent studies using metabolomics have shown that there are differences in metabolic fingerprints between these two groups [9, 10].

Metabolomics is a new, rapidly expanding field of systems biology with the ability to measure all small molecules, chemicals, and metabolites that can be identified in a given sample comprehensively [11, 12]. Metabolomics is based on high-throughput approaches, which separate the population of unknown small molecules for subsequent quantification and identification, rather than quantifying a molecule of known identity [13]. The resolution of compounds using gas chromatography (GC) and liquid chromatography (LC) has provided significant benefits as compared with two-dimensional gel electrophoresis [13]. Mass spectrometry (MS) and nuclear magnetic resonance (NMR) spectroscopy have promoted accuracy and sensitivity for identifying unknown metabolites [14]. Such an untargeted approach allows acquisition of the global metabolite profile in a biological compartment, without any prior hypothesis, facilitated by a blind comparison between cases and controls [15]. Nevertheless, limitations in quantitative measurements and metabolite annotations remain problematic in untargeted metabolomics [16]. On the other hand, a targeted metabolomics approach measures and analyzes metabolites in known or predicted metabolic pathways. Analytical methods for targeted metabolomics can be optimized and their quantitative results should be more reliable than those of untargeted metabolomics [16].

The aim of this study was to find diagnostic metabolites that distinguish sepsis-induced ARDS patients from non-ARDS controls using this targeted metabolomics approach, and to identify metabolites and related pathways that can differentiate sepsis-induced direct and indirect ARDS. A targeted metabolomics strategy, focusing on energy metabolism, free fatty acids, amino acids, sphingolipids, and phospholipids were chosen because it has been reported that these metabolites might have some connection to metabolic alterations in ARDS [17–22].

## Methods

### Study design and patient selection

This study retrospectively analyzed the samples of the ASAN sepsis registry obtained from March 2011 to February 2018, along with the non-ARDS controls. This study was approved by the Asan Institutional Review Board (IRB No. 2019–1017). The need for obtaining informed consent was waived because the study used an existing cohort sample.

The inclusion criteria for the cohort were as follows: adult patients aged > 18 years, patients admitted to the medical intensive care unit (ICU) and clinically diagnosed with sepsis according to the Sepsis-2 definition [23], who were enrolled within 48 h of ICU admission. The patients' serum samples were collected at the time of sepsis cohort enrollment. The non-ARDS control group was enrolled from the registry of individuals who visited the Health Screening and Promotion Center at Asan Medical Center for a health screening. Among them, those without acute diseases such as infection and no obvious abnormalities on chest radiography and similar age and sex to the ARDS patients were selected as the control group. At the time of blood collection, they were in good health and had no special symptoms or signs, and even if there was an underlying disease, the condition was well controlled.

The definition of ARDS followed the Berlin definition based on PaO_2_/FiO_2_[24]. The diagnosis of direct ARDS and indirect ARDS was made through the consensus in blinded review by two intensivists. The following ARDS patients were excluded: (1) patients who underwent chemotherapy within the last month, (2) patients who were administered immunosuppressants after organ transplantation, (3) patients who had drug-induced pneumonitis, (4) patients in an immunocompromised state, and (5) patients whose diagnosis of ARDS could not be agreed upon by the two intensivists. Since the eligible ARDS patients belonged to the sepsis cohort, the direct ARDS group included pulmonary ARDS patients with pneumonia-induced sepsis, and the indirect ARDS included extrapulmonary ARDS patients with non-pneumonia-induced sepsis.

### Data collection

Comprehensive clinical data were collected on the first day of admission to ICU, including severity of illness scores, such as the Acute Physiology and Chronic Health Evaluation II (APACHE II) and Sequential Organ Failure Assessment (SOFA), co-morbidities, laboratory values, and source of infection. Thereafter, clinical outcomes were collected, including 28-day mortality data.

### Targeted metabolomics approach

Samples of the patients with sepsis-induced direct ARDS and indirect ARDS as well as those of the non-ARDS controls were analyzed and quantified using targeted metabolomics. Metabolites involved in energy metabolism (glycolysis, citric acid cycle, and the pentose phosphate pathway) as well as those in free fatty acid, amino acid, phospholipid, and sphingolipid metabolism were measured. Laboratory analysis was conducted using LC–tandem MS (LC–MS/MS) and GC–MS systems. Six batches were used for this study, and the same number of samples from each group were allocated across the batches. Pooled human plasma samples (Sigma-Aldrich) were used for quality control (QC), and two QC runs were included in each batch. The analysis order was randomized among the samples in a batch. Several runs with blank samples, standard solutions, and QC samples were performed to check the robustness of the analytical method before study sample analysis. Metabolic features with CV < 20% in QC samples were considered acceptable. Principal component analysis (PCA) score plot for serum metabolome data including QC samples was shown in Additional file 1: Fig. S1. A detailed description of the methodology is presented separately in the Supplemental Materials. Target metabolome data are publicly available at Metabolomics Workbench (StudyID: ST002550, ST002702).

### Data processing

Data were further processed with normalization, scaling, filtering (removing metabolic features with 50% missing values), and statistical analysis using MetaboAnalyst 5.0 (www.metaboanalyst.ca), a web server designed for comprehensive analysis of metabolomics data for visualization and interpretation. Datasets were normalized, log-transformed, and auto-scaled to generate more comparable individual features prior to the statistical analyses. Missing values were estimated by replacement with small values (half of the minimum positive value in the original data).

### Statistical analysis

When comparing clinical and baseline characteristics between groups, continuous variables are reported as medians (interquartile range, 25–75%), and categorical variables are reported as numbers (percentages). Data were compared for continuous variables using Wilcoxon’s rank-sum test when comparing the medians of two groups. Pearson’s χ^2^ test or Fisher’s exact test were used to compare categorical variables. Pearson’s correlation analysis or Spearman’s correlation analysis was used for normally distributed and non-normally distributed data, when checking the correlation between patient demographics and metabolites. Significance was defined by *p* < 0.05. For all analyses, a two-tailed *p*-value < 0.05 was considered statistically significant. Statistical analysis was performed using IBM SPSS version 25.0 (IBM Corp., Armonk, NY, USA). To identify differentially expressed metabolites among the compared groups (e.g., ARDS vs. controls; direct vs. indirect ARDS), principal component analysis (PCA), partial least-squares discriminant analysis (PLS-DA) were performed using MetaboAnalyst 5.0. We also performed analysis of covariance for each significant metabolite to adjust demographical characteristics such as age, gender and body mass index (BMI), and comorbidities such as chronic liver disease, chronic kidney disease or malignant disease. Metabolic pathway analysis was performed using the pathway analysis program in the MetaboAnalyst 5.0 to recognize important biological pathways in sepsis-induced ARDS and subphenotypes. Each metabolite term was converted to a known human matabolome database (HMDB) ID via HMDB identifier. Then, quantitative pathway analysis was performed by grouping differently with the concentration data of all metabolites as follows: i) ARDS vs non-ARDS controls, ii) Direct ARDS vs indirect ARDS, iii) non-ARDS controls vs direct ARDS, and iv) non-ARDS controls vs indirect ARDS. Pathways with the false discovery rate (FDR) *p*-value of less than 0.05 were considered significant.

## Results

### Patients’ characteristics

Eighty-four patients were included, of which 54 were patients with sepsis-induced ARDS and 30 were non-ARDS controls. The sepsis-induced ARDS patients consisted of 27 patients each with direct and indirect subphenotypes. The baseline characteristics of all ARDS patients and non-ARDS controls are provided in Table 1. The proportions of co-morbidities such as chronic liver disease, solid tumor malignancy, and neurologic disease were significantly different between the two groups. A comparison of the baseline and clinical characteristics of direct ARDS and indirect ARDS groups is presented in Table 2. Patients with direct ARDS were significantly older than those with indirect ARDS. A BMI was significantly higher in the patients with indirect ARDS. There were no statistically significant difference in co-morbidities between the two groups although respiratory disease was more common in direct ARDS. Initial APACHE II score was similar between the groups, whereas patients with indirect ARDS had a significantly higher SOFA score, along with a higher rate of bacteremia and higher lactate levels, than did those with direct ARDS.

**Table 1:** Baseline characteristics of sepsis-induced ARDS patients at ICU admission compared with non-ARDS controls

Characteristics	ARDS patients	Non-ARDS controls	*p*-value
Number of patients	54	30	
Male (n, %)	36 (67)	20 (67)	1.000
Age	68 [57–76]	63 [62–64]	0.546
BMI (kg/m^2^)	23.6 [20.8–26.2]	23.8 [22.3–25.5]	0.079
Co-morbidities			
Respiratory disease	8 (15)	1 (4)	0.483
Cardiovascular disease	3 (6)	1 (3)	1.000
Neurological disease	7 (13)	0 (0)	0.047
Chronic kidney disease	2 (4)	0 (0)	0.535
Chronic liver disease	12 (22)	0 (0)	0.003
Diabetes	10 (19)	5 (17)	0.832
Malignancy			
Solid tumor	22 (41)	0 (0)	< 0.001
Hematologic	1 (2)	0 (0)	1.000

**Table 2:** Patient clinical and baseline characteristics at ICU admission according to sepsis-induced ARDS subphenotype

Characteristics	Direct ARDS	Indirect ARDS	*p*-value
Number of patients	27	27	
Male (n, %)	19 (70)	17 (63)	0.564
Age	74 [64–79]	64 [55–71]	0.004
BMI (kg/m^2^)	22.3 [20.0–24.9]	26.1 [22.0–28.3]	< 0.001
Co-morbidities			
Respiratory disease	7 (26)	1 (4)	0.050
Cardiovascular disease	3 (11)	0 (0)	0.236
Neurological disease	6 (22)	1 (4)	0.100
Chronic kidney disease	0 (0)	2 (7)	0.491
Chronic liver disease	5 (19)	7 (26)	0.513
Diabetes	4 (15)	6 (22)	0.484
Malignancy			
Solid tumor	10 (37)	12 (44)	0.580
Hematologic	0 (0)	1 (4)	1.000
Source of infection			< 0.001
Pneumonia	27 (100)	0 (0)	
Intraabdominal	0 (0)	12 (44)	
Soft tissue	0 (0)	5 (19)	
Urinary tract	0 (0)	4 (15)	
Others	0 (0)	6 (22)	
Presence of bacteremia	7 (26)	21 (78)	< 0.001
Identification of pathogen	17 (63)	22 (82)	0.129
Bacteria	16 (89)	22 (100)	0.436
Virus	1 (6)	0 (0)	
APACHE II score	25 [22–29]	27 [23–29]	0.205
SOFA score	10 [9–12]	14 [11-17]	< 0.001
Severity of ARDS			0.355
Mild	5 (19)	6 (22)	
Moderate	10 (37)	14 (52)	
Severe	12 (44)	7 (26)	
Invasive MV	26 (96)	24 (89)	0.610
Laboratory findings			
P/F ratio at diag. of ARDS	109 [72–148]	128 [85–202]	0.197
Lactate	3.5 [2.0–6.8]	6.4 [2.9–11.0]	0.025
WBC (10^3^/mm^3^)	8.9 [5.1–12.2]	21.2 [6.4–24.9]	0.013
Platelet (10^3^/mm^3^)	144 [104–190]	45 [12–109]	< 0.001
BUN (mg/dL)	29 [17-46]	39 [24-27]	0.170
CRP (mg/dL)	18.4 [11.7–33.5]	20.3 [7.8–25.6]	0.504
Procalcitonin (ng/mL)	1.70 [0.26–17.04]	28.77 [6.70–192.99]	0.011
28-day mortality	11 (41)	15 (56)	0.276

Direct ARDS = Pneumonia sepsis-induced ARDS, Indirect ARDS = Non-pneumonia sepsis-induced ARDS, MV = Mechanical Ventilation, APACHE II = Acute Physiology and Chronic Health Evaluation-II, P/F ratio = PaO_2_/FiO_2_ ratio, SOFA = Sequential Organ Failure Assessment

### ARDS vs controls

**Fig. 1:**
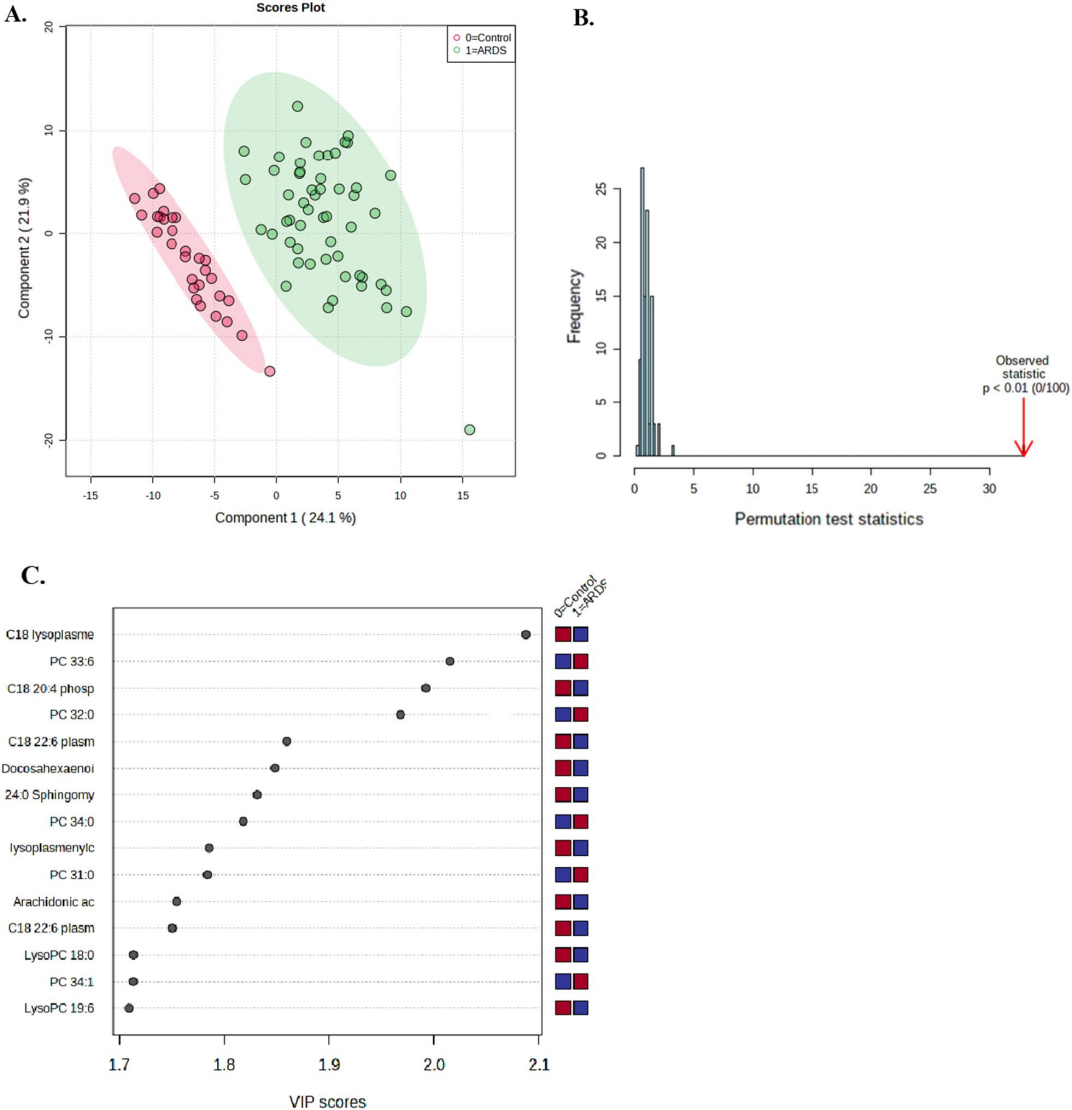

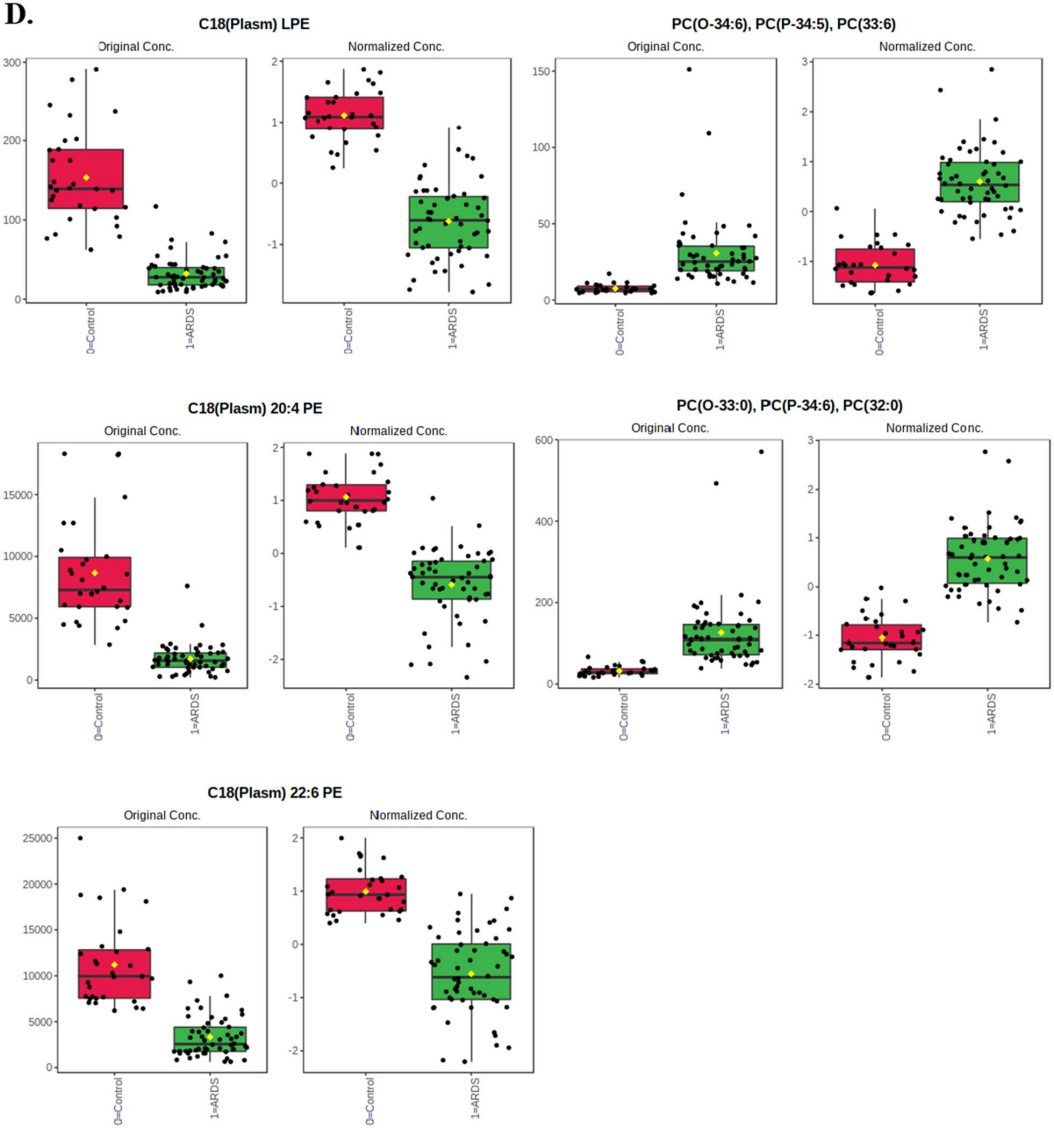
Statistical analysis of the data obtained for acute respiratory distress syndrome (ARDS) patients and non-ARDS controls. **A** Partial least squares discriminant analysis (PLS-DA) showing the separation of sepsis-induced ARDS patients (green) from non-ARDS controls (red). **B** Permutation test statistics using separation distance based on sum of squares between and sum of squares within (B/W) ratio. This test indicates PLS-DA between ARDS patients and non-ARDS controls was statistically significant (*p* < 0.01). **C** Variable importance in projection (VIP) score. The metabolites responsible for discrimination between ARDS patients and non-ARDS controls are shown. Metabolites with high VIP scores are more important in class separation. **D** Concentrations of significant metabolites for the discrimination of sepsis-induced ARDS and non-ARDS controls

A total of 186 metabolites, including 16 metabolites involved in energy metabolism, 8 involved in free fatty acid, 32 involved in amino acid, 115 involved in phospholipid (including 8 plasmalogens), and 15 involved in sphingolipid metabolism were identified by LC–MS/MS or GC–MS in the 84 subjects. On comparing the overall sepsis-induced ARDS patient group (n = 54) with the non-ARDS control group (n = 30), marked differences were observed in 102 compounds in all measured metabolite classes (Additional file 1: Table S1). We chose 5 components which was achieved by cross validation method of PLS-DA with R^2^ = 0.96, Q^2^ = 0.91, and accuracy of 1.0 (Fig. 1C and Additional file 1: Fig. S2). Then, we selected top 5 components based on a variable important in projection (VIP) score (> 1.0) as metabolites distinguishing sepsis-induced ARDS from non-ARDS controls, which were from lysophosphatidylethanolamine (lysoPE) plasmalogen (C18 (Plasm) lysoPE), phosphatidylethanolamine plasmalogen (C18(Plasm) 20:4 PE, C18(Plasm) 22:6 PE), and phosphatidylcholine (PC (33:6), PC (32:0)) metabolism. These 5 most significant metabolites were derived from phospholipid metabolism, and among them, three PE plasmalogens showed a significantly lower concentration in the sepsis-induced ARDS group than in the non-ARDS control group. On the other hand, the two PCs were significantly higher in the sepsis-induced ARDS than in the non-ARDS control group (Fig. 1D). These metabolites were still significant in distinguishing ARDS vs non-ARDS controls after adjusting the effect of other factors such as age, gender, BMI, and the presence of chronic liver disease, chronic kidney disease or malignant disease.

### Metabolic pathway analysis for distinguishing ARDS from controls

**Fig. 2:**
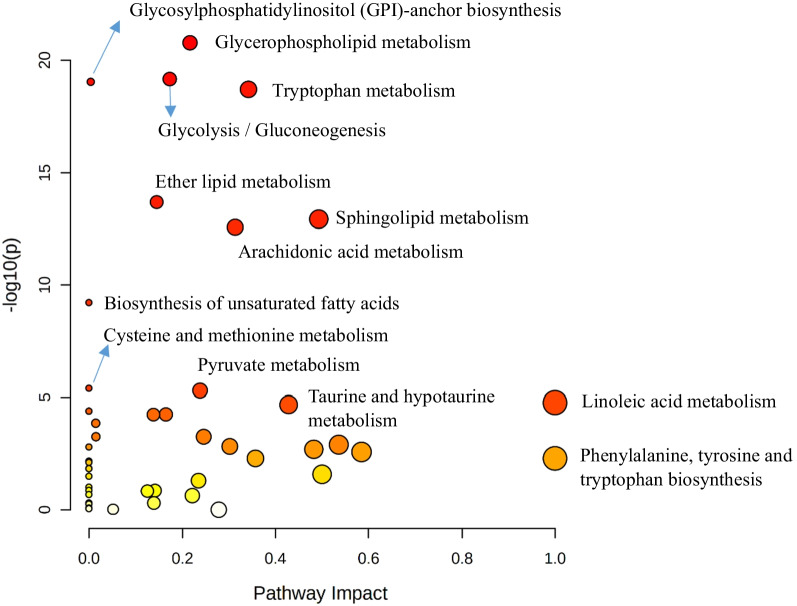
Pathways affected in sepsis-induced ARDS biology. *Color gradient and circle size indicate the significance of the pathway ranked by *p*-value (yellow: higher *p*-value, red: lower *p*-value) and pathway impact score (larger circle indicates higher impact score)

The top-10 pathways significantly associated with sepsis-induced ARDS were 1) glycerophospholipid metabolism (FDR = 8.44 × 10^–20^), 2) glycolysis/gluconeogenesis (FDR = 1.52 × 10^–18^), 3) glycosylphosphatidylinositol (GPI)-anchor biosynthesis (FDR = 1.52 × 10^–18^), 4) tryptophan metabolism (FDR = 2.48 × 10^–18^), 5) ether lipid metabolism (FDR = 2.01 × 10^–13^), 6) sphingolipid metabolism (FDR = 9.65 × 10^–13^), 7) arachidonic acid metabolism (FDR = 1.89 × 10^–12^), 8) biosynthesis of unsaturated fatty acids (FDR = 3.80 × 10^–9^), 9) cysteine and methionine metabolism (FDR = 2.23 × 10^–5^), and 10) pyruvate metabolism (FDR = 2.52 × 10^–5^) (Fig. 2, Additional file 1: Table S2).

### Direct ARDS vs indirect ARDS as ARDS subphenotypes

**Fig. 3:**
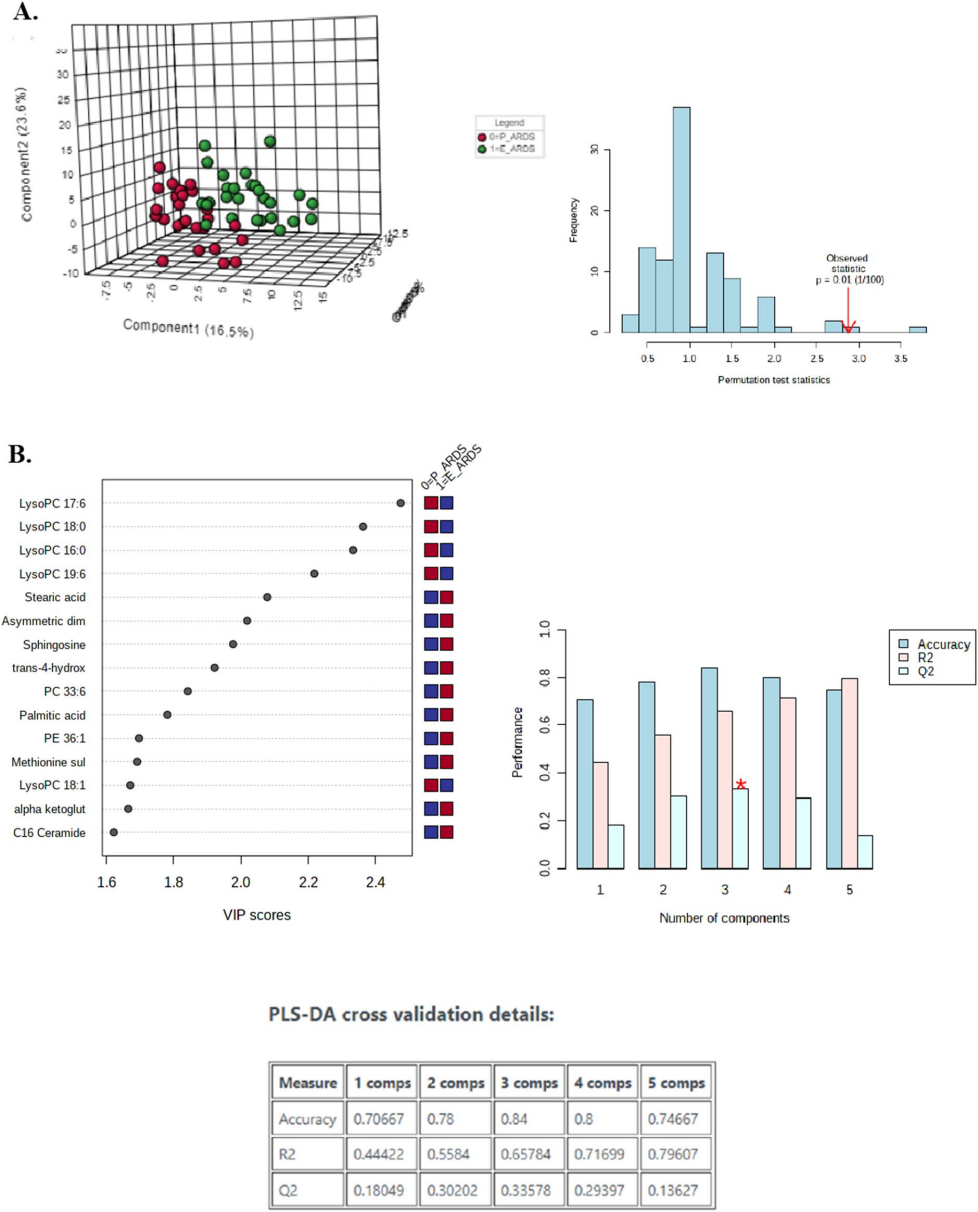

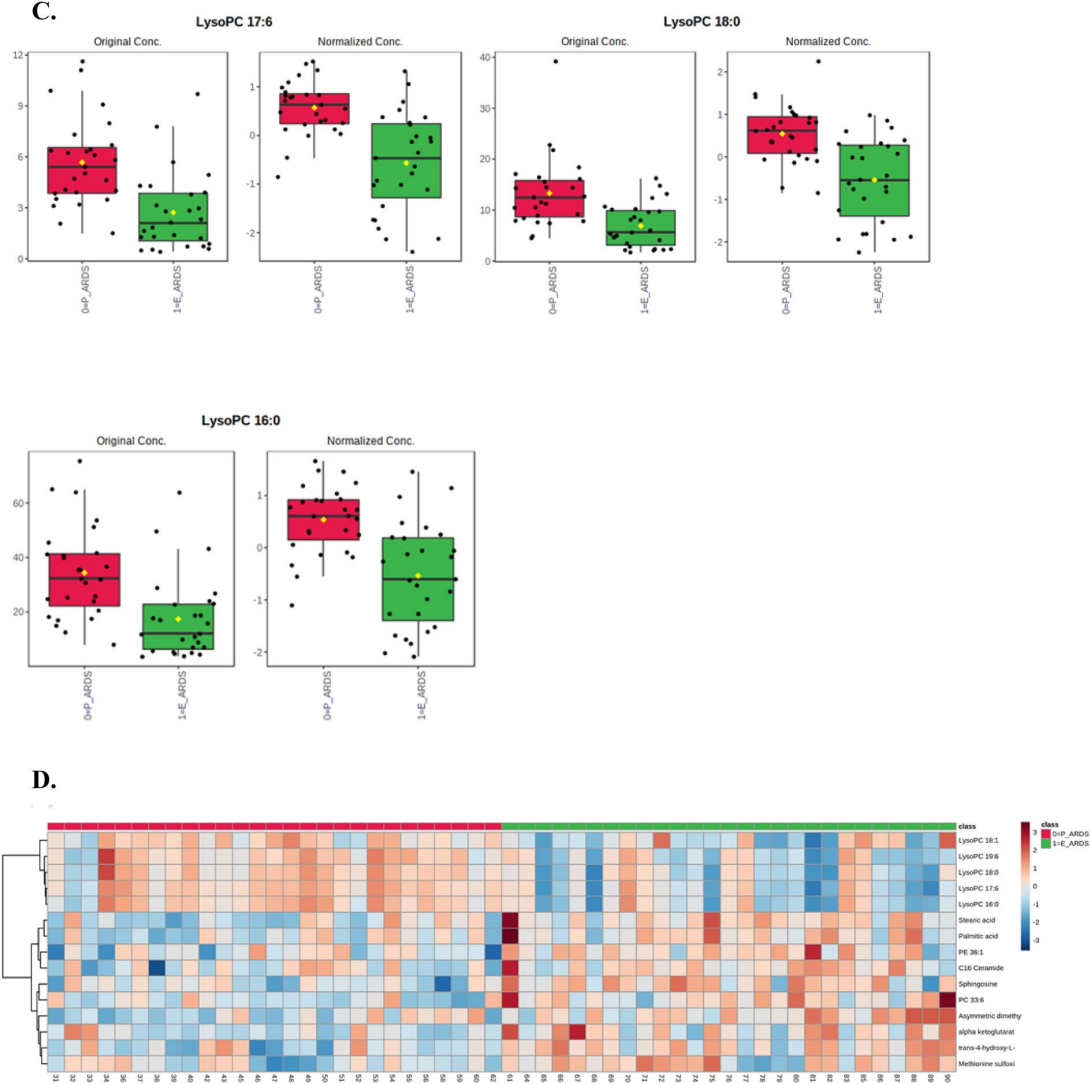
Statistical analysis of the data obtained for 54 patients with 27 direct ARDS and 27 indirect ARDS. **A** PLS-DA 3D score plot for the discrimination of patients with sepsis-induced direct ARDS (P-ARDS) and indirect ARDS (E-ARDS) (left) and permutation test (right) indicating that the PLS-DA between ARDS patients and non-ARDS controls was statistically significant (p = 0.01). **B** Important metabolites discriminating the two groups. Variable importance in projection (VIP) score: the metabolites are responsible for discrimination between direct ARDS and indirect ARDS. Metabolites with high VIP scores are more important in class separation. **C** Concentrations of significant metabolites for the discrimination of sepsis-induced direct ARDS and indirect ARDS. **D** Hierarchical heatmap for top-15 discriminating metabolites between sepsis-induced direct ARDS and indirect ARDS (red bar: direct ARDS, green bar: indirect ARDS)

When analyzing the sepsis-induced direct and indirect ARDS groups using PLS-DA, 3-dimensional score plotting showed that the two groups were distinguished by metabolic profiles (Fig. 3A). We chose 3 components which was achieved by cross validation method of PLS-DA with R^2^ = 0.66, Q^2^ = 0.3, and accuracy of 0.84. Then, we selected 3 metabolites to discriminate sepsis-induced direct and indirect ARDS, which were lysoPC (17:6), lysoPC (18:0), and lysoPC (16:0) among 14 significantly different metabolites shown in Table 3. Higher concentrations of lysoPC (16:0), lysoPC (17:6), and lysoPC (18:0) were observed in the direct ARDS group as compared to the indirect ARDS group (Fig. 3C). A heatmap using Pearson’s correlation and Ward’s linkage shows the intuitive visualization of discriminant metabolites between the sepsis-induced direct and indirect ARDS subgroups (Fig. 3D). These three lysoPCs were still significant after adjusting for age, gender, BMI, and the presence of chronic liver disease, chronic kidney disease or malignant disease.

**Table 3:** Significantly different serum metabolites between sepsis-induced direct ARDS and indirect ARDS

Metabolites	t.stat	p.value	− log10(p)	FDR
LysoPC 17:6	5.0578	5.62E−06	5.2499	0.0009843
LysoPC 18:0	4.7275	1.77E−05	4.7513	0.0013795
LysoPC 16:0	4.6435	2.36E−05	4.6262	0.0013795
LysoPC 19:6	4.3284	6.85E−05	4.1641	0.0029982
Stearic acid	−3.9689	0.000223	3.6527	0.0077874
Asymmetric dimethylarginine	−3.8218	0.000356	3.449	0.010373
Sphingosine	−3.7214	0.000487	3.312	0.012187
Trans-4-hydroxy-L-proline	−3.5918	0.000728	3.1377	0.01593
PC 33:6	−3.4088	0.001268	2.8969	0.024654
Palmitic acid	−3.2729	0.001895	2.7224	0.033165
PE 36:1	−3.0885	0.003225	2.4915	0.048624
Methionine sulfoxide	−3.0768	0.003334	2.477	0.048624
LysoPC 18:1	3.0328	0.003775	2.4231	0.048903
Oxoglutaric acid	−3.02	0.003912	2.4076	0.048903

FDR = false discovery rate, LysoPC = lysophosphatidylcholine, PC = phosphatidylcholine**,** PE = phosphatidylethanolamine

### Metabolic pathways discriminating between direct ARDS and indirect ARDS

Pathway analysis of serum metabolites was performed in sepsis-induced ARDS subphenotypes. The pathways most involved in direct and indirect ARDS were different. Compared to non-ARDS controls, the top-5 pathways associated with sepsis-induced direct ARDS were 1) GPI-anchor biosynthesis (FDR = 7.17 × 10^–16^) 2) glycerophospholipid metabolism (FDR = 1.13 × 10^–16^), 3) tryptophan metabolism (FDR = 2.77 × 10^–14^), 4) sphingolipid metabolism (FDR = 1.1 × 10^–13^), and 5) biosynthesis of unsaturated fatty acids (FDR = 2.6 × 10^–10^) (Additional file 1: Fig. S3A). Also, compared to non-ARDS controls, the top-5 pathways affected in sepsis-induced indirect ARDS were (1) sphingolipid metabolism (FDR = 6.3 × 10^–16^), (2) glycerophospholipid metabolism (FDR = 8.0 × 10^–16^), (3) tryptophan metabolism (FDR = 2.7 × 10^–13^), (4) GPI-anchor biosynthesis (FDR = 7.8 × 10^–13^), and (5) ether lipid metabolism (FDR = 9.9 × 10^–13^) (Additional file 1: Fig. S3B). Among the top 10 pathways of each ARDS subphenotype, the common or exclusive pathways were analyzed. Direct ARDS was more related to fatty acid metabolisms such as biosynthesis of unsaturated fatty acids and fatty acid degradation, while indirect ARDS was more related to energy metabolisms such as glycolysis/gluconeogenesis and pyruvate metabolism (Additional file 1: Fig. S3C).

**Fig. 4:**
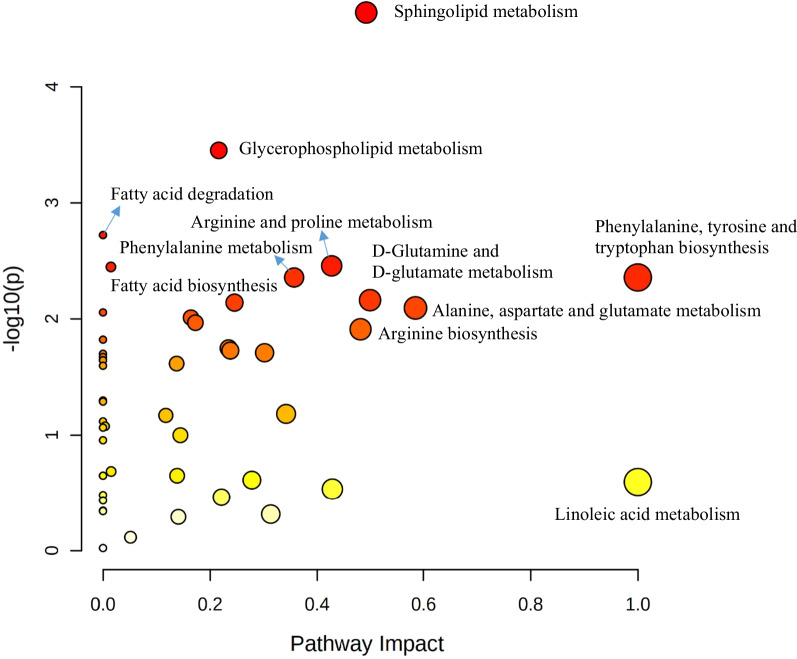
Metabolic pathway analysis significantly discriminating between direct acute respiratory distress syndrome (ARDS) and indirect ARDS. *Color gradient and circle size indicate the significance of the pathway ranked by *p*-value (yellow: higher *p*-value, red: lower *p*-value) and pathway impact score (larger circle indicates higher impact score)

Significant pathways discriminating sepsis-induced direct ARDS and indirect ARDS are presented in Fig. 4. The top distinguishing pathways were (1) sphingolipid metabolism (FDR = 1.15 × 10^–3^), (2) glycerophospholipid metabolism (FDR = 8.83 × 10^–3^), (3) arginine and proline metabolism (FDR = 2.7 × 10^–2^), (4) fatty acid biosynthesis (FDR = 2.7 × 10^–2^), 5) phenylalanine, tyrosine, and tryptophan biosynthesis (FDR = 2.7 × 10^–2^), and 6) phenylalanine metabolism (FDR = 2.7 × 10^–2^).

### Sphingolipid metabolism as a key pathway discriminating between direct and indirect ARDS

**Fig. 5:**
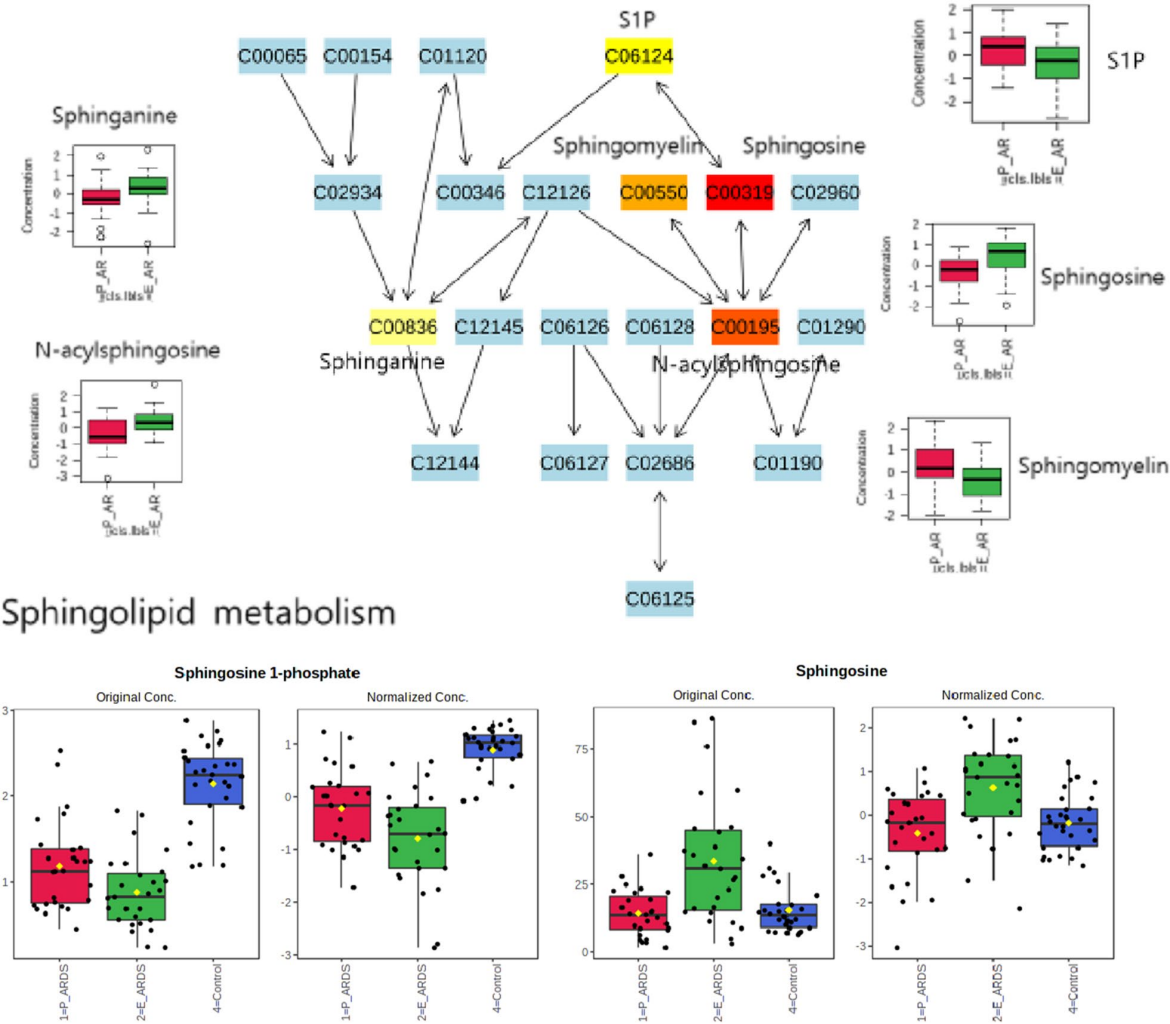
Sphingolipid metabolic pathway involving acute respiratory distress syndrome (ARDS) subphenotypes. *Sepsis-induced direct ARDS (P-ARDS) vs. sepsis-induced indirect ARDS (E-ARDS)

Sphingolipid metabolism was the most significant pathway for differentiating between sepsis-induced direct and indirect ARDS. Among the related metabolites, sphingosine-1-phosphate (S1P) and sphingosine were distinctive. S1P was markedly lower in both sepsis-induced ARDS groups as compared with the non-ARDS control group (Fig. 5), and was also lower in the indirect ARDS than in the direct ARDS group, although this was not statistically significant. Sphingosine showed considerably different concentrations between the two groups, with significantly higher levels in indirect ARDS than in direct ARDS patients.

## Discussion

In this study, we investigated the differences in the metabolome and related pathways between sepsis-induced ARDS and non-ARDS controls, and between sepsis patients with direct and with indirect ARDS as a subphenotype, using LC–MS/MS and GC–MS for targeted metabolomics. Among 186 metabolites detected, 102 metabolites could differentiate sepsis-induced ARDS patients from the non-ARDS controls, and 14 metabolites could discriminate between the direct and indirect ARDS subphenotypes. In PLS-DA, we found that sepsis-induced ARDS was metabolically distinct from the non-ARDS controls, and that sepsis-induced direct and indirect ARDS were also metabolically different subgroups.

We identified significant differences in sepsis-induced ARDS patients and non-ARDS controls in terms of metabolites related to ARDS biology. The main substances involved were PE plasmalogens, including C18(Plasm) lysoPE, C18(Plasm) 20:4 PE, and C18(Plasm) 22:6, which were decreased in patients as compared to controls. Plasmalogens are plasma-borne antioxidant phospholipid species that provide protection as cellular lipid components during cellular oxidative stress [25]. According to a previous human study, plasmalogens were decreased in the bronchoalveolar lavage (BAL) fluid of an ARDS patient [26]. In a recent study that evaluated the serum lipid profile of COVID-19 patients, it was confirmed that PE plasmalogens were significantly decreased as the P/F ratio decreased [27]. In sepsis patients, plasmalogens also decreased, which suggested that these molecules may be a marker of oxidative stress [28]. Recently, it was found that PE plasmalogens were decreased further in moderate to severe COVID-19 disease, and among the relevant metabolites, the decrease in PE (P-18:0/20:4) and PE (P-18:0/22:6) was consistent with our results [29].

It had not previously been clear how PC levels change in ARDS. The concentrations of PC (33:6) and PC (32:0) were increased in sepsis-induced ARDS patients as compared to non-ARDS controls. However, according to a recent study, plasma PC concentrations, mainly in the PC (18:2) series, were significantly lower in ARDS patients than in normal controls, which was thought to be due to abnormal changes in PC synthesis in the liver of ARDS patients [30]. However, in our results, PC (33:6) and PC (32:0) levels were higher in sepsis-induced ARDS patients than in the controls, suggesting that the direction of increase or decrease may be different for each PC series. While several studies have found an increase in PC levels in sepsis patients [31–33], other studies have shown a decrease in these patients as compared to normal controls [31, 32, 34, 35]. In one study, the PC (34:3) level was significantly lower in patients with pneumonia as a primary focus of sepsis than in patients with other primary foci, such as intra-abdominal, urinary tract, or blood stream infections. On the other hand, a high PC (34:1) level was shown to be a prognostic marker suggestive of septic shock in a pneumonia group [31]. These results suggest that PC species containing long-chain fatty acids can be important metabolites in sepsis-induced lung injury or ARDS.

We confirmed that the metabolites that showed the most significant difference between sepsis-induced direct and indirect ARDS were lysoPCs, including lysoPC (17:6), lysoPC (18:0), and lysoPC (16:0). The concentrations of lysoPC in the sepsis-induced ARDS groups were significantly decreased as compared with the non-ARDS control group, while the direct ARDS group showed higher levels than did the indirect ARDS group. LysoPC is a lipid mediator derived from membrane PC, which has been suggested to regulate immune responses. PC is hydrolyzed by phospholipase A_2_ (PLA_2_), resulting in the production of lysoPC [36]. LysoPC is known to contribute to inflammation by increasing chemokine production and activating endothelium, neutrophils, monocytes, macrophages, and lymphocytes [37]. However, the role of lysoPCs in ARDS has not yet been clearly elucidated. Although there were differences in the change in lysoPC levels in each study, the enzymes involved in this process have been suggested to be biomarkers for ARDS or acute lung injury [38]. In a preclinical study on the activity of type II PLA_2_, the concentration of lysoPC in BAL fluid was higher than that of controls with a higher activity of type II PLA_2_ [39]. In clinical studies, PLA_2_ activity in BAL fluid and plasma of patients were also increased as compared to controls [40, 41]. Additionally, when comparing direct and indirect ARDS, PLA_2_ activity was higher in direct ARDS cases [40]. On the other hand, studies of sepsis patients showed the opposite results. In sepsis patients, serum lysoPC levels were lower than those in controls, and among them, lysoPC (16:0) and (18:0) were decreased [42]. Lower concentrations of serum or plasma lysoPC predicted worse outcomes [43], and the ratio of lysoPC/PC was lower in sepsis patients than in healthy controls [42]. In our study, the decrease in serum lysoPCs was similar to that seen in sepsis. However, higher concentrations in direct than in indirect ARDS patients were consistent with previous studies showing increased results of lysoPC (16:0) and lysoPC (18:0) in lung diseases with epithelial damage [44]. That is, our results appear to be primarily based on biological changes in sepsis because our study patients had ARDS based on sepsis. But, since they showed metabolic differences following direct or indirect lung injury, the corresponding lysoPCs could be important markers for differentiating between the two subphenotypes of ARDS.

We identified that sphingolipid metabolism is an important pathway for distinguishing between sepsis-induced direct and indirect ARDS patients, and confirmed that there was a difference in S1P. S1P is a naturally occurring bioactive sphingolipid generated by sphingomyelin metabolism [45]. It is generated by the phosphorylation of sphingosine, catalyzed by sphingosine kinases (SphKs) 1 and 2, and is catabolized by lipid phosphate phosphatases, S1P phosphatases, and S1P lyase [45]. S1P plays an important role in the vascular and immune systems [46]. In acute lung injury, S1P has been recognized as a potent angiogenic factor enhancing lung endothelial integrity and inhibiting vascular permeability [45, 47]. Previous studies showed different results, depending on whether it was a preclinical or clinical study [48–52]. Preclinical studies showed upregulation of S1P in lung tissues, BAL fluid, and plasma in cases with acute lung injury [48–50], whereas a clinical study showed lower serum S1P levels in ARDS patients [52], which was consistent with our results. In addition, we observed a distinct pattern between direct and indirect ARDS at the level of sphingosine. In indirect ARDS patients, sphingosine was upregulated as compared to non-ARDS controls or direct ARDS patients. This result suggested that the following three mechanisms can be a clue to distinguishing between indirect and direct ARDS: 1) activation of S1P phosphatases, which are rate limiting enzymes for reversing S1P into sphingosine; 2) inactivation of SphKs, which convert sphingosine to S1P; or 3) upregulation of the S1P receptor. However, this will require further research, as we could not confirm the activity of these enzymes in each group.

Since there was a difference in the underlying disease between the ARDS patients and the non-ARDS controls, we analyzed whether the presence of co-morbidities such as chronic liver disease and solid tumor affected metabolic differences. Thus, we performed PLS-DA for ARDS patients and control individuals without chronic liver disease, and found that metabolites such as C18(Plasm) LPE, PC(33:6), C18(Plasm) 20:4 PE, and PC(32:0) were still included among the top differential metabolites between the ARDS patients and the control group. Also, when we ran the analysis again by excluding those with a solid tumor, the main 4 metabolites that distinguished ARDS from non-ARDS controls remained in the top metabolites. In the ARDS subphenotype analysis (direct vs. indirect), there was no significant difference in the co-morbidities between the groups, except for the higher BMI in the indirect group. However, we assume that BMI would not have significantly affected the distinguishing metabolites for two reasons. Firstly, the proportion of patients in the indirect ARDS group who were actually obese (BMI ≥ 30 kg/m^2^) was low; secondly, a correlation analysis between BMI and distinguishing metabolites indicated that the value of the correlation coefficient was very small, albeit a significant negative correlation between them. However, because the number of patients in our study was small, it is necessary to confirm this with more patients in the future.

Our study shares similarities with the Metwaly study [9], as both compare ARDS and control groups using metabolomics, analyze direct and indirect ARDS, and present associated metabolic pathways and biomarkers. A significant commonality between the two studies is that the ARDS group in both cases was sourced from the sepsis network. Glycerophospholipid and tryptophan metabolism were consistently identified as metabolic pathways associated with ARDS in both studies. Additionally, the finding that the primary pathway of indirect ARDS is related to energy metabolism was consistent across both studies. However, there were several differences between the two studies. First, the metabolomics methodology varied; the Metwaly study employed an untargeted approach using ^1^H-NMR spectroscopy and GC–MS, while our study used a targeted approach with LC–MS/MS and GC–MS. Second, the blood sampling time differed. In our study, a single blood collection for the ARDS group occurred within 48 h after ICU admission, while the Metwaly study collected samples on ICU admission day 1 and tracked them temporally in some recovered patients. Third, the study controls were distinct; the Metwaly study used ICU-ventilated patients unrelated to lung diseases as controls, while our study's controls were subjects who visited medical institutions for health checkups without acute disease or chest radiography abnormalities. Lastly, the ARDS patient characteristics varied. Our study's patients were older, predominantly male, had higher severity at ICU admission, and experienced higher 28-day mortality than those in the Metwaly study. Despite these differences, both studies share key findings: ARDS is metabolically distinct compared to the control group, and ARDS subphenotypes exhibit clear differences.

Our results align with a recent study by Alipanah-Lechner et al. [53], which differentiated ARDS into hyperinflammatory and hypoinflammatory subtypes. This study, like Metwaly's [9], focused on sepsis patients with ARDS, making its patient group quite similar to ours. The hyperinflammatory and hypoinflammatory subtypes in their study closely resembled the indirect and direct ARDS subphenotypes in our research. Patients with hyperinflammatory ARDS exhibited significantly lower plasma lipid concentrations and increased levels of glycolytic metabolites, such as lactate and pyruvate, compared to those with hypoinflammatory ARDS. Similarly, in our study, patients with indirect ARDS had lower lipid levels (primarily very long-chain fatty acids, lysophosphatidylcholines, and some sphingomyelins) and higher levels of glycolytic metabolism. Our findings also revealed that the primary metabolic pathways involved in indirect ARDS were glycolysis/gluconeogenesis metabolism and pyruvate metabolism, which supports this observation. These overlaps between the results of the two studies suggest that the clinically different ARDS subphenotypes are consistent with metabolically distinct subtypes.

The present study had several limitations. First, we collected a sample only at one time point, at ICU admission. The serial change patterns of metabolites are related to the prognosis of ARDS [9], but we could not confirm this. Second, although blood samples were taken as early as possible on the first day of diagnosis, there could be differences between the onset of disease and time of diagnosis. Thus, some metabolites may have been limited in terms of their presence in the serum. Third, since all of our study patients had infections, the interpretation of our results is limited as to whether these findings are specific to ARDS. Further validation of the identified metabolites and pathways is required, and additional research on ARDS caused by non-infectious etiologies is necessary.

In conclusion, despite these limitations, our study demonstrated a marked difference in the metabolic pattern between sepsis-induced ARDS and non-ARDS controls. We also identified that direct ARDS and indirect ARDS are metabolically distinct subphenotypes. In particular, lysoPC (17:6), lysoPC (16:0), and lysoPC (18:0) of glycerophospholipid metabolism and S1P of sphingomyelin metabolism demonstrated potential as important markers for subphenotype distinction. This study provides a basis for further research into the development of theranostics based on these metabolites.

## Supplementary Information

**Additional file 1.** Methodology, Supplementary tables and figures.

## Data Availability

The data sets generated and/or analyzed during the study are not publicly available but may be available from the corresponding author upon reasonable request.

## Abbreviations

APACHE II: Acute Physiology and Chronic Health Evaluation II
ARDS: Acute respiratory distress syndrome
BAL: Bronchoalveolar lavage
BMI: Body mass index
FDR: False discovery rate
GC-MS: Gas chromatography-mass spectrometry
GPI: Glycosylphosphatidylinositol
ICU: Intensive care unit
LC–MS/MS: Liquid chromatography-tandem mass spectrometry
LysoPC: Lysophosphatidylcholine
LysoPE: Lysophosphatidylethanolamine
PC: Phosphatidylcholine
PE: Phosphatidylethanolamine
PLS-DA: Partial least-squares discriminant analysis
PLA2: Phospholipase A2
SOFA: Sequential Organ Failure Assessment
SphK: Sphingosine kinase
S1P: Sphingosine-1-phosphate
