# Supplementary material for: A targeted metabolomics approach for sepsis-induced ARDS and its subphenotypes
Source: Crit Care. 2023 Jul 5;27:263. doi: 10.1186/s13054-023-04552-0 (PMC10320874; doi:10.1186/s13054-023-04552-0)
Supplement: Supplementary file 1 — Additional file 1. Methodology, Supplementary tables and figures. [file 13054_2023_4552_MOESM1_ESM.docx]

**SUPPLEMENTARY MATERIALS**

1. Methodology

2. Supplementary tables and figures

**1. Methodology**

**Laboratory analysis**

**1) Metabolites related to energy metabolism, amino acids, ceramide, sphingomyelin, plasmalogen, phosphatidylcholine, and phosphatidylethanolamine**

***Materials***

Metabolite standards and internal standards were purchased from Sigma–Aldrich (St Louis, MO, USA) and Avanti Polar Lipids (Alabaster, AL, USA). All solvents, including water, were purchased from J. T. Baker Chemicals (Mumbai, India). For amino acids, the internal standards and derivatization reagents were purchased from Sigma–Aldrich or CDN Isotopes Inc. (Pointe-Claire, QC, Canada).

***Sample Preparation***

To 50 μL of human serum, we added 50 μL of internal standard solutions (10 μM ^13^C_5_-glutamine, 0.4 μM serotonine-d_4_, 0.6 μM dopamine-d_4_, and 2 μM tryptophan-d_5_ for energy metabolism- and amino acid-related analyses; 50 nM C17 ceramide for ceramide-related analysis; 200 nM 18:0 D70 phosphatidylinositol (PC) and 1 μM 16:0 D31-18:1 phosphatidylethanolamine (PE) for plasmalogen and PC/PE profiling) were added. Metabolites were extracted from aqueous and organic phases by liquid–liquid extraction. Sample solutions were prepared by using commonly used liquid-liquid extaction procedure known as Bligh/Dyer mthod with minor modifications (Can.J.Biochem.Physiol. 37:911-917). Briefly, 400 μL of chloroform/methanol (1/2, v/v) was added to each sample solution and mixed well. The solution was centrifuged for 15 min at 14000 rpm. After centrifugation, a thick precipitate containing macromolecules was found between the aqueous upper layer and the organic lower layer. Nonpolar metabolites containing lipids were contained in the lower organic phase, and polar metabolites were contained in the upper aqueous phase. We carefully withdrew ~90% of each phase, making sure to avoid interface. The collected volume from each layer were generally the same, however sometimes any specific sample had thicker interface between organic and aqueous layer, which resulted in little variation of the recovered volumes. However, internal standards added prior to sample preparation should correct this variation.The aqueous and organic phases were dried under vacuum and stored at -20℃ until further analysis. The dried matter from the aqueous phase was reconstituted with 50 μL of H_2_O/MeOH (50/50 v/v) and the dried matter from organic phase was reconstituted with MeOH, prior to liquid chromatography–tandem mass spectrometry (LC-MS/MS) analysis. For amino acids, 10 μL out of the total 50 μL reconstituted aqueous phase was used for chemical derivatization of amino acids using phenylisothiocyanate. The remaining 40 μL reconstituted aqueous phase was used to measure metabolites of energy metabolism. After the reaction, the derivatized amino acids were extracted with 100 μL of 5 mM ammonium acetate in MeOH and were then subjected to LC-MS/MS analysis.

***Liquid Chromatography–Tandem Mass Spectrometry***

Metabolites were analyzed with an LC-MS/MS device equipped with a 1290 high-performance liquid chromatography device (HPLC; Agilent Technologies, Santa Clara, CA, USA), Qtrap 5500 (ABSciex, Framingham, MA, USA), and an LC column. For each type of analysis, 3 μL of each sample solution was injected into the LC-MS/MS system and ionized with a turbo spray ionization source.

For metabolites related to energy metabolism, a Synergi fusion-RP (50 × 2 mm; Phenomenex, Torrance, CA, USA) HPLC column was used. As mobile phases A and B, 5 mM ammonium acetate in H_2_O and 5 mM ammonium acetate in MeOH were used, respectively. The separation gradient was as follows: hold at 0% B for 5 min, 0% to 90% B for 2 min, hold at 90% for 8 min, 90% to 0% B for 1 min, then hold at 0% B for 9 min. The LC flow rate was 70 μL/min except for 140 μL/min during minutes 7–15, and the column temperature was kept at 23°C. Multiple reaction monitoring (MRM) was used in negative ion mode.

For amino acid-related analyses, a Zorbax Eclipse XDB-C18 (100 × 2mm; Agilent Technologies) column was used. As mobile phases A and B, 0.2% formic acid in H_2_O and 0.2% formic acid in acetonitrile were used, respectively. The separation gradient was as follows: hold at 0% B for 0.5 min, 0–95% B for 5 min, 95% B for 1 min, and 95–0% B for 0.5 min, then hold at 0% B for 2.5 min. The LC flow rate was 500 μL/min, and the column temperature was kept at 50°C. MRM was used in positive ion mode.

For ceramide and sphingomyelin, a Pursuit5 C18 (150 × 2.1 mm; Agilent Technologies) was used with mobile phase A (5 mM ammonium formate/MeOH/tetrahydrofuran, 500/200/300) and mobile phase B (5 mM ammonium formate/MeOH/ tetrahydrofuran, 100/200/700). The LC rate was 200 µL/min, and the column temperature was kept at 35°C. The LC gradient was as follows: 50% A for 0 min, 50% A for 5 min, 50–30% A for 3 min, 30% A for 7 min, 30–10% A for 7 min, 10% A for 3 min, 10–50% A for 0.1 min, and 50% A for 4.9 min. MRM was used in positive ion mode.

For plasmalogen, a Zorbax Eclipse C18 (50 × 2.1 mm) was used with mobile phase A (10 mM ammonium acetate in MeOH/isopropanol/H2O, 900/50/50) and mobile phase B (10 mM ammonium acetate in MeOH/isopropanol/H2O, 940/50/10). The LC run was performed with the isocratic condition of 60% B at 400 µl/min for 20 min. MRM was used in positive ion mode.

For PC and PE profiling, flow injection analysis–tandem mass spectrometry (FIA-MS/MS) was performed. The same mobile phases as in the ceramide analysis were used to profile PC (precursor ion scan of 184) and PE (neutral loss scan of 141) with the isocratic condition of 50% B at 70 µl/min for 3 min. Extracted ion chromatograms were reconstructed with masses corresponding to each PC or PE species and were used for quantitation.

Data analysis was performed using Analyst 1.5.2 software (Sciex). Extracted ion chromatograms (EIC) corresponding to each metabolite were used for quantitation. The area under the curve of each EIC was normalized to that of the internal standard. For metabolites related to energy metabolism and PC/PE profiling, the peak area ratio of each metabolite to that of the internal standard was used for a relative comparison. For amino acid-related analyses, the calibration curves ranged from 1 nM to 600 μM, with R^2^ > 0.98. For ceramide- and plasmalogen-related analyses, the calibration range was generally 0.1 nM to 10 μM, with R^2^ > 0.99.

**2) Sphingosine-1-phosphate**

***Sample preparation***

50 μL of human serum were mixed well with 50 μl internal standard solution (1 μM C17 sphingosine-1-phosphate [S1P] solution) before extraction. Then, 75 μL of chloroform, 50 μL of MeOH, 12.5 μL of H_2_O, and 2.5 μL of 10 N NaOH were added to each sample and mixed well. After centrifugation, the upper alkaline phase was collected. Next, 100 μL of chloroform and 5 μL of 10 N HCl were added to the alkaline phase and the samples were mixed well. After phase separation by centrifugation, the lower organic phase was collected. The organic solutions containing S1P were dried under vacuum and stored at -20°C until LC-MS/MS analysis. The dried matter was reconstituted with MeOH and injected into the LC-MS/MS system. The lipid standards were purchased from Avanti Polar Lipids.

***Liquid chromatography-tandem mass spectrometry***

Lipid levels were determined using an LC-MS/MS system equipped with an Ultimate3000 HPLC (Dionex, Bayern, Germany) and OrbitrapXL (ABSciex, Toronto, ON, Canada). A reverse-phase column (Jupiter 5-µm C4, 50 × 1.0 mm) was used with mobile phase A (0.1% formic acid in H_2_O) and mobile phase B (0.1% formic acid in MeOH). The LC was run at 300 µL/min and 35°C. The LC gradient was as follows: 10% B for 0 min, 10–90% B for 5 min, 90% B for 10 min, 90–10% B for 0.1 min, and 10% B for 4.9 min. Extracted ion chromatograms corresponding to the specific transition (phosphate/S1P) was used for quantification in the negative ion mode. Data analysis was performed using Xcaliber 2.2 software (ThermoFisher Scientific; Waltham, MA, USA).

**3) Free fatty acids**

***Sample preparation***

We mixed 50 μL of human serum with 200 μL of cold MeOH and 50 μL of internal standard solution (0.1 mg/mL myristic acid-d_14_). Glass vials were used for preparing sample preparation. Sample solutions were acidified with HCl to a 25 mM final concentration and centrifuged. Supernatants were collected into fresh tubes, and 4 mL of iso-octane were added. The upper phase was collected after the liquid–liquid extraction process and then dried under vacuum. The dried sample was reacted with 200 μL of BCl_3_-MeOH and 12% w/w (Sigma–Aldrich) at 60°C for 30 min. Next, 100 μL of H_2_O and 100 μL of hexane were added sequentially, and the sample was mixed vigorously. The upper phase was collected after resting the sample for 5 min. Then, 10–20 mg of anhydrous sodium sulfate were added, at which point the supernatant was ready for gas chromatography–mass spectrometry (GC-MS) analysis. Commercially available fatty acid methyl esters (Sigma–Aldrich) were used to generate calibration curves without derivatization.

***Gas chromatography-mass spectrometry***

Fatty acid methyl esters were analyzed with a GC-MS system (Agilent 7890A/5975C) using a capillary column (HP-5MS, 30 m × 0.25 mm × 0.2 µm). Electron impact (EI) ionization was used in positive ion mode. A 1-μL injection volume and split mode (ratio 10:1) were used. The total analysis time was 73.7 min, and the temperature gradient was as follows: hold at 50°C for 2 min, 50–120°C at 10°C/min, 120–250°C at 3°C/min, hold at 250°C for 15 min, 250–300°C at 35°C/min, hold at 300°C for 5 min. Extracted ion chromatograms, according to the specific fragment ion for each fatty acid, were used for quantification. Data analysis was performed using MSD Chemstation software (Agilent E02.02.1431).

**2. Supplementary tables and figures**

**Supplementary Table 1. Metabolites that differed significantly between the sepsis-induced ARDS patients and non-ARDS control.**

| **Metabolites** | **t.stat** | **p-value** | **-log10(p)** | **FDR** |
| --- | --- | --- | --- | --- |
| C18(Plasm)LPE | 13.839 | 3.71E-23 | 22.43 | 6.50E-21 |
| PC 33:6 | -12.407 | 1.64E-20 | 19.784 | 1.44E-18 |
| C18(Plasm)20:4 PE | 12.014 | 9.12E-20 | 19.04 | 5.32E-18 |
| PC 32:0 | -11.624 | 5.08E-19 | 18.294 | 2.22E-17 |
| C18(Plasm)22:6 PE | 10.123 | 4.26E-16 | 15.37 | 1.49E-14 |
| Docosahexaenoic acid | 9.9877 | 7.89E-16 | 15.103 | 2.30E-14 |
| 24:0 SM | 9.7879 | 1.96E-15 | 14.707 | 4.90E-14 |
| PC 34:0 | -9.6372 | 3.90E-15 | 14.409 | 8.53E-14 |
| C18(Plasm) LPC | 9.2789 | 2.01E-14 | 13.698 | 3.79E-13 |
| PC 31:0 | -9.2624 | 2.16E-14 | 13.665 | 3.79E-13 |
| Arachidonic acid | 8.9602 | 8.64E-14 | 13.064 | 1.37E-12 |
| C18(Plasm) 22:6 PC | 8.9164 | 1.06E-13 | 12.977 | 1.54E-12 |
| LysoPC 18:0 | 8.5556 | 5.51E-13 | 12.259 | 6.94E-12 |
| PC 34:1 | -8.554 | 5.55E-13 | 12.255 | 6.94E-12 |
| LysoPC 19:6 | 8.5152 | 6.63E-13 | 12.178 | 7.74E-12 |
| S1P | 8.2634 | 2.10E-12 | 11.678 | 2.30E-11 |
| PC 33:1 | -8.0648 | 5.20E-12 | 11.284 | 5.35E-11 |
| PC 33:0 | -7.929 | 9.66E-12 | 11.015 | 9.39E-11 |
| Tryptophan | 7.8827 | 1.19E-11 | 10.924 | 1.10E-10 |
| C24 Ceramide | 7.8608 | 1.32E-11 | 10.88 | 1.15E-10 |
| Kynurenine | -7.8265 | 1.54E-11 | 10.813 | 1.28E-10 |
| C18(Plasm)18:1 PE | 7.8019 | 1.72E-11 | 10.764 | 1.37E-10 |
| PC 32:1 | -7.7484 | 2.20E-11 | 10.658 | 1.67E-10 |
| PC 32:6 | -7.7243 | 2.45E-11 | 10.611 | 1.79E-10 |
| LysoPC 16:0 | 7.3642 | 1.25E-10 | 9.9028 | 8.75E-10 |
| PC 30:0 | -7.204 | 2.57E-10 | 9.5897 | 1.73E-09 |
| 18:1 SM | 7.0775 | 4.54E-10 | 9.3434 | 2.94E-09 |
| C16 Ceramide | -6.9543 | 7.87E-10 | 9.1043 | 4.92E-09 |
| LysoPC 18:2 | 6.9281 | 8.84E-10 | 9.0535 | 5.33E-09 |
| LysoPC 17:6 | 6.8277 | 1.38E-09 | 8.8595 | 8.06E-09 |
| PC 31:6 | -6.7733 | 1.76E-09 | 8.7546 | 9.93E-09 |
| PC 30:1 | -6.7445 | 2.00E-09 | 8.6993 | 1.09E-08 |
| C18(Plasm) 20:4 PC | 6.4759 | 6.52E-09 | 8.1855 | 3.46E-08 |
| LysoPC 18:1 | 6.3377 | 1.19E-08 | 7.9233 | 6.14E-08 |
| PC 30:2 | -6.2618 | 1.66E-08 | 7.7801 | 8.30E-08 |
| PC 31:1 | -6.1959 | 2.21E-08 | 7.6562 | 1.07E-07 |
| PC 34:2 | -6.1079 | 3.23E-08 | 7.4914 | 1.53E-07 |
| PC 42:0 | -6.0809 | 3.62E-08 | 7.441 | 1.67E-07 |
| PC 35:0 | -6.0386 | 4.34E-08 | 7.3621 | 1.95E-07 |
| PC 36:0 | -5.9872 | 5.41E-08 | 7.2666 | 2.37E-07 |
| Glucose | -5.9797 | 5.59E-08 | 7.2528 | 2.38E-07 |
| PC 36:1 | -5.9404 | 6.61E-08 | 7.1798 | 2.75E-07 |
| Serotonin | 5.7551 | 1.45E-07 | 6.8388 | 5.90E-07 |
| 16:0 SM | 5.73 | 1.61E-07 | 6.7928 | 6.29E-07 |
| PC 42:1 | -5.729 | 1.62E-07 | 6.7911 | 6.29E-07 |
| PC 35:6 | -5.7059 | 1.78E-07 | 6.7488 | 6.78E-07 |
| Lactate | -5.6701 | 2.07E-07 | 6.6836 | 7.71E-07 |
| 18:0 SM | 5.6356 | 2.39E-07 | 6.6209 | 8.73E-07 |
| PC 37:0 | 5.1546 | 1.73E-06 | 5.7631 | 6.16E-06 |
| 24:1 SM | 5.1462 | 1.78E-06 | 5.7484 | 6.25E-06 |
| PC 30:3 | -5.0529 | 2.59E-06 | 5.586 | 8.90E-06 |
| Pyruvate | -4.943 | 4.01E-06 | 5.3964 | 1.35E-05 |
| PC 36:2 | -4.8689 | 5.37E-06 | 5.2697 | 1.77E-05 |
| Linoleic acid | 4.788 | 7.37E-06 | 5.1326 | 2.39E-05 |
| PC 29:0 | -4.7382 | 8.94E-06 | 5.0486 | 2.85E-05 |
| Stearic acid | 4.6573 | 1.22E-05 | 4.9132 | 3.82E-05 |
| Taurine | 4.5184 | 2.07E-05 | 4.6835 | 6.36E-05 |
| C18(Plasm)18:1 PC | 4.3786 | 3.50E-05 | 4.456 | 0.000106 |
| Palmitic acid | 4.3461 | 3.95E-05 | 4.4038 | 0.000117 |
| PC 29:1 | -4.1745 | 7.40E-05 | 4.131 | 0.000216 |
| PE 36:1 | -4.0428 | 0.000119 | 3.9258 | 0.00034 |
| PC 35:1 | -4.0275 | 0.000125 | 3.9022 | 0.000354 |
| PC 39:0 | 3.9314 | 0.000176 | 3.7553 | 0.000488 |
| Acetylornithine | 3.656 | 0.000451 | 3.3458 | 0.001233 |
| Phenylalanine | -3.6327 | 0.000488 | 3.312 | 0.001313 |
| Methionine sulfoxide | -3.5768 | 0.000587 | 3.2314 | 0.001556 |
| Oleic acid | 3.5518 | 0.000637 | 3.1956 | 0.001665 |
| Malate | -3.5448 | 0.000652 | 3.1856 | 0.001679 |
| Asymmetric dimethylarginine | -3.5119 | 0.000726 | 3.1388 | 0.001843 |
| Citrulline | 3.5018 | 0.000751 | 3.1245 | 0.001877 |
| PC 43:4 | -3.4356 | 0.000931 | 3.0313 | 0.002294 |
| PC 31:2 | -3.417 | 0.000988 | 3.0053 | 0.002401 |
| PC 43:6 | -3.3844 | 0.001096 | 2.96 | 0.002607 |
| PC 38:2 | -3.3813 | 0.001107 | 2.9557 | 0.002607 |
| PC 29:2 | -3.3784 | 0.001118 | 2.9518 | 0.002607 |
| PE 36:2 | -3.3057 | 0.001407 | 2.8518 | 0.00324 |
| Serine | 3.2895 | 0.00148 | 2.8296 | 0.003344 |
| PC 34:3 | -3.2873 | 0.001491 | 2.8267 | 0.003344 |
| PC 43:5 | -3.2609 | 0.001619 | 2.7908 | 0.003586 |
| PC 35:2 | -3.1667 | 0.002165 | 2.6645 | 0.004737 |
| symmetric dimethylarginine | -3.1388 | 0.002358 | 2.6274 | 0.005095 |
| Myristic acid | 2.9993 | 0.003583 | 2.4458 | 0.007608 |
| PC 38:6 | 2.9949 | 0.00363 | 2.4401 | 0.007608 |
| Leucine | 2.9928 | 0.003652 | 2.4375 | 0.007608 |
| PC 34:4 | 2.9438 | 0.004217 | 2.375 | 0.008599 |
| Isoleucine | 2.943 | 0.004226 | 2.3741 | 0.008599 |
| Fumarate | -2.8888 | 0.004945 | 2.3059 | 0.009946 |
| PC 41:4 | -2.8644 | 0.005305 | 2.2754 | 0.010549 |
| PC 36:6 | 2.8489 | 0.005546 | 2.2561 | 0.010904 |
| C18 Ceramide | -2.8299 | 0.005853 | 2.2326 | 0.011381 |
| PC 38:5 | 2.7704 | 0.006923 | 2.1597 | 0.013314 |
| PC 28:1 | -2.7613 | 0.007103 | 2.1485 | 0.013512 |
| PC 33:2 | -2.7536 | 0.007257 | 2.1392 | 0.013656 |
| PC 39:3 | -2.7433 | 0.00747 | 2.1267 | 0.013906 |
| Succinate | -2.5078 | 0.014122 | 1.8501 | 0.026015 |
| Glutamine | 2.4985 | 0.014469 | 1.8396 | 0.026376 |
| PC 42:6 | 2.4183 | 0.017807 | 1.7494 | 0.032125 |
| PC 41:5 | -2.412 | 0.018097 | 1.7424 | 0.032316 |
| PC 35:3 | -2.3957 | 0.018864 | 1.7244 | 0.033346 |
| Palmitoleic acid | 2.3691 | 0.020181 | 1.6951 | 0.035317 |
| PC 28:0 | -2.3016 | 0.023897 | 1.6217 | 0.041406 |
| PE 38:4 | -2.263 | 0.026277 | 1.5804 | 0.045083 |

**Supplementary Table 2. List of the metabolites used for pathway analysis.**

| **Metabolites** | **HMDB ID** | **P-value** | **FDR** |
| --- | --- | --- | --- |
| C18(Plasm)LPE | N/A | 3.71E-23 | 6.53E-21 |
| PC 33:6 | N/A | 1.64E-20 | 1.45E-18 |
| C18(Plasm)20:4 PE | HMDB0005779 | 9.12E-20 | 5.35E-18 |
| PC 32:0 | HMDB0000564 | 5.08E-19 | 2.23E-17 |
| C18(Plasm)22:6 PE | HMDB0011394 | 4.26E-16 | 1.50E-14 |
| Docosahexaenoic acid | HMDB0002183 | 7.89E-16 | 2.32E-14 |
| 24:0 SM | HMDB0011697 | 1.96E-15 | 4.93E-14 |
| PC 34:0 | HMDB0007878 | 3.90E-15 | 8.58E-14 |
| C18(Plasm) LPC | HMDB0011149 | 2.01E-14 | 3.81E-13 |
| PC 31:0 | HMDB0007935 | 2.16E-14 | 3.81E-13 |
| Arachidonic acid | HMDB0001043 | 8.64E-14 | 1.38E-12 |
| C18(Plasm) 22:6 PC | HMDB0011262 | 1.06E-13 | 1.55E-12 |
| LysoPC 18:0 | HMDB0010384 | 5.51E-13 | 6.98E-12 |
| PC 34:1 | HMDB0007879 | 5.55E-13 | 6.98E-12 |
| LysoPC 19:6 | N/A | 6.63E-13 | 7.78E-12 |
| S1P | HMDB0000277 | 2.10E-12 | 2.31E-11 |
| PC 33:1 | HMDB0007938 | 5.20E-12 | 5.38E-11 |
| PC 33:0 | HMDB0007937 | 9.66E-12 | 9.44E-11 |
| Tryptophan | HMDB0000929 | 1.19E-11 | 1.10E-10 |
| C24 Ceramide | HMDB0004956 | 1.32E-11 | 1.16E-10 |
| Kynurenine | HMDB0000684 | 1.54E-11 | 1.29E-10 |
| C18(Plasm)18:1 PE | HMDB0011375 | 1.72E-11 | 1.38E-10 |
| PC 32:1 | HMDB0007872 | 2.20E-11 | 1.68E-10 |
| PC 32:6 | N/A | 2.45E-11 | 1.80E-10 |
| LysoPC 16:0 | HMDB0010382 | 1.25E-10 | 8.80E-10 |
| PC 30:0 | HMDB0007869 | 2.57E-10 | 1.74E-09 |
| 18:1 SM | HMDB0012101 | 4.54E-10 | 2.96E-09 |
| C16 Ceramide | HMDB0004949 | 7.87E-10 | 4.94E-09 |
| LysoPC 18:2 | HMDB0010386 | 8.84E-10 | 5.37E-09 |
| LysoPC 17:6 | N/A | 1.38E-09 | 8.11E-09 |
| PC 31:6 | N/A | 1.76E-09 | 9.99E-09 |
| PC 30:1 | HMDB0007870 | 2.00E-09 | 1.10E-08 |
| C18(Plasm) 20:4 PC | HMDB0011253 | 6.52E-09 | 3.48E-08 |
| LysoPC 18:1 | HMDB0002815 | 1.19E-08 | 6.18E-08 |
| PC 30:2 | HMDB0007903 | 1.66E-08 | 8.34E-08 |
| PC 31:1 | HMDB0007936 | 2.21E-08 | 1.08E-07 |
| PC 34:2 | HMDB0007880 | 3.23E-08 | 1.53E-07 |
| PC 42:0 | HMDB0008058 | 3.62E-08 | 1.68E-07 |
| PC 35:0 | HMDB0007944 | 4.34E-08 | 1.96E-07 |
| PC 36:0 | HMDB0007886 | 5.41E-08 | 2.38E-07 |
| Glucose | HMDB0000122 | 5.59E-08 | 2.40E-07 |
| PC 36:1 | HMDB0007887 | 6.61E-08 | 2.77E-07 |
| Serotonin | HMDB0000259 | 1.45E-07 | 5.93E-07 |
| 16:0 SM | HMDB0010169 | 1.61E-07 | 6.33E-07 |
| PC 42:1 | HMDB0008059 | 1.62E-07 | 6.33E-07 |
| PC 35:6 | N/A | 1.78E-07 | 6.82E-07 |
| Lactate | HMDB0000190 | 2.07E-07 | 7.76E-07 |
| 18:0 SM | HMDB0001348 | 2.39E-07 | 8.78E-07 |
| PC 37:0 | HMDB0007953 | 1.73E-06 | 6.20E-06 |
| 24:1 SM | HMDB0012107 | 1.78E-06 | 6.28E-06 |
| PC 30:3 | N/A | 2.59E-06 | 8.95E-06 |
| Pyruvate | HMDB0000243 | 4.01E-06 | 1.36E-05 |
| PC 36:2 | HMDB0000593 | 5.37E-06 | 1.78E-05 |
| Linoleic acid | HMDB0000673 | 7.37E-06 | 2.40E-05 |
| PC 29:0 | HMDB0007868 | 8.94E-06 | 2.86E-05 |
| Stearic acid | HMDB0000827 | 1.22E-05 | 3.84E-05 |
| Taurine | HMDB0000251 | 2.07E-05 | 6.40E-05 |
| C18(Plasm)18:1 PC | HMDB0011243 | 3.50E-05 | 0.000106 |
| Palmitic acid | HMDB0000220 | 3.95E-05 | 0.000118 |
| PC 29:1 | HMDB0007901 | 7.40E-05 | 0.000217 |
| PE 36:1 | HMDB0008993 | 0.000119 | 0.000342 |
| PC 35:1 | HMDB0007945 | 0.000125 | 0.000356 |
| PC 39:0 | HMDB0007959 | 0.000176 | 0.000491 |
| Acetylornithine | HMDB0012175 | 0.000451 | 0.00124 |
| Phenylalanine | HMDB0000159 | 0.000488 | 0.00132 |
| Methionine sulfoxide | HMDB0002005 | 0.000587 | 0.001565 |
| Oleic acid | HMDB0000207 | 0.000637 | 0.001674 |
| Malate | HMDB0000156 | 0.000652 | 0.001688 |
| Asymmetric dimethylarginine | HMDB0001539 | 0.000726 | 0.001853 |
| Citrulline | HMDB0000904 | 0.000751 | 0.001888 |
| PC 43:4 | N/A | 0.000931 | 0.002307 |
| PC 31:2 | N/A | 0.000988 | 0.002415 |
| PC 43:6 | N/A | 0.001096 | 0.002622 |
| PC 38:2 | HMDB0007927 | 0.001107 | 0.002622 |
| PC 29:2 | N/A | 0.001118 | 0.002622 |
| PE 36:2 | HMDB0008994 | 0.001407 | 0.003258 |
| Serine | HMDB0062263 | 0.00148 | 0.003363 |
| PC 34:3 | HMDB0007881 | 0.001491 | 0.003363 |
| PC 43:5 | N/A | 0.001619 | 0.003607 |
| PC 35:2 | HMDB0007946 | 0.002165 | 0.004764 |
| symmetric dimethylarginine | HMDB0003334 | 0.002358 | 0.005124 |
| Myristic acid | HMDB0000806 | 0.003583 | 0.007651 |
| PC 38:6 | HMDB0007991 | 0.00363 | 0.007651 |
| Leucine | HMDB0000687 | 0.003652 | 0.007651 |
| PC 34:4 | HMDB0007883 | 0.004217 | 0.008648 |
| Isoleucine | HMDB0000172 | 0.004226 | 0.008648 |
| Fumarate | HMDB0000134 | 0.004945 | 0.010003 |
| PC 41:4 | N/A | 0.005305 | 0.010609 |
| PC 36:6 | HMDB0007892 | 0.005546 | 0.010966 |
| C18 Ceramide | HMDB0004950 | 0.005853 | 0.011446 |
| PC 38:5 | HMDB0007989 | 0.006923 | 0.01339 |
| PC 28:1 | HMDB0007867 | 0.007103 | 0.013589 |
| PC 33:2 | HMDB0007940 | 0.007257 | 0.013734 |
| PC 39:3 | N/A | 0.00747 | 0.013985 |
| Succinate | HMDB0000254 | 0.014122 | 0.026163 |
| Glutamine | HMDB0000148 | 0.014469 | 0.026527 |
| PC 42:6 | HMDB0008288 | 0.017807 | 0.032309 |
| PC 41:5 | N/A | 0.018097 | 0.0325 |
| PC 35:3 | HMDB0007947 | 0.018864 | 0.033536 |
| Palmitoleic acid | HMDB0003229 | 0.020181 | 0.035519 |
| PC 28:0 | HMDB0007866 | 0.023897 | 0.041643 |
| PE 38:4 | HMDB0009003 | 0.026277 | 0.04534 |
| C18:1 Ceramide | HMDB0004948 | 0.033154 | 0.056651 |
| Nicotinamide Adenine Dinucleotide | HMDB0000902 | 0.037004 | 0.062623 |
| PC 36:5 | HMDB0007890 | 0.038696 | 0.064861 |
| Valine | HMDB0000883 | 0.039113 | 0.064943 |
| PC 32:2 | HMDB0007874 | 0.041689 | 0.068573 |
| PC 38:3 | HMDB0008020 | 0.046607 | 0.075953 |
| Glutamate | HMDB0000148 | 0.049416 | 0.079792 |
| Nicotinamide Adenine Dinucleotide Phosphate | HMDB0000217 | 0.050158 | 0.080252 |
| Arginine | HMDB0000517 | 0.06292 | 0.099765 |
| Threonine | HMDB0000167 | 0.081946 | 0.12877 |
| PC 40:2 | HMDB0008053 | 0.091287 | 0.14218 |
| PC 40:6 | HMDB0008057 | 0.1075 | 0.16502 |
| PE 38:5 | HMDB0011358 | 0.10851 | 0.16502 |
| PC 39:2 | N/A | 0.10876 | 0.16502 |
| PC 41:1 | N/A | 0.11095 | 0.16689 |
| PC 40:5 | HMDB0008055 | 0.11617 | 0.17327 |
| PC 34:6 | HMDB0007918 | 0.13238 | 0.19579 |
| C24:1 Ceramide | HMDB0004953 | 0.13864 | 0.20271 |
| alpha-aminoadipic acid | HMDB0000510 | 0.13936 | 0.20271 |
| PC 37:4 | HMDB0007955 | 0.15165 | 0.21726 |
| C20 Ceramide | HMDB0004951 | 0.15183 | 0.21726 |
| PC 42:2 | HMDB0008092 | 0.17419 | 0.24724 |
| Proline | HMDB0000162 | 0.20065 | 0.28251 |
| PC 28:2 | HMDB0007900 | 0.20821 | 0.29083 |
| PC 32:3 | HMDB0007875 | 0.21085 | 0.2922 |
| Sphingosine | HMDB0000252 | 0.21659 | 0.29782 |
| PC 36:3 | HMDB0007921 | 0.22228 | 0.30185 |
| PC 37:3 | N/A | 0.22296 | 0.30185 |
| PC 37:5 | HMDB0007956 | 0.23981 | 0.32219 |
| Ornithine | HMDB0000214 | 0.24366 | 0.32477 |
| PC 40:1 | HMDB0008052 | 0.24542 | 0.32477 |
| PE 40:6 | HMDB0011392 | 0.25285 | 0.33211 |
| PC 38:4 | HMDB0007988 | 0.29153 | 0.38007 |
| PC 39:4 | N/A | 0.30329 | 0.3925 |
| PC 39:6 | N/A | 0.31985 | 0.41081 |
| trans-4-hydroxy-L-proline | HMDB0000725 | 0.32211 | 0.41081 |
| PC 40:0 | HMDB0008051 | 0.33593 | 0.42535 |
| PC 40:3 | HMDB0008086 | 0.35776 | 0.44976 |
| PC 40:4 | HMDB0008054 | 0.37348 | 0.46618 |
| PC 37:6 | HMDB0007958 | 0.4178 | 0.51783 |
| PC 35:4 | HMDB0007949 | 0.43725 | 0.53816 |
| PC 38:1 | HMDB0007894 | 0.44278 | 0.54118 |
| PC 39:1 | HMDB0007960 | 0.46707 | 0.56693 |
| Glucose-6-phosphate / Fructose-6-phosphate | N/A | 0.49829 | 0.60067 |
| Aspartate | HMDB0000191 | 0.51853 | 0.62082 |
| Spermidine | HMDB0001257 | 0.52687 | 0.62655 |
| PC 41:3 | N/A | 0.54859 | 0.648 |
| Tyrosine | HMDB0000158 | 0.55557 | 0.65186 |
| PC 36:4 | HMDB0007889 | 0.58331 | 0.67988 |
| Citrate / Iso citrate | N/A | 0.60249 | 0.69762 |
| PC 41:2 | N/A | 0.60899 | 0.70053 |
| PE 38:6 | HMDB0009102 | 0.61411 | 0.70184 |
| C14 Ceramide | HMDB0011773 | 0.62387 | 0.7084 |
| PC 41:0 | N/A | 0.63008 | 0.71086 |
| Ribose-1,5-bisphosphate | HMDB0011688 | 0.64068 | 0.71822 |
| Glycine | HMDB0000123 | 0.69089 | 0.7696 |
| PC 38:0 | HMDB0007893 | 0.74116 | 0.81356 |
| Sedoheptulose-7-phosphate | HMDB0001068 | 0.7431 | 0.81356 |
| PC 39:5 | N/A | 0.74422 | 0.81356 |
| PC 33:3 | HMDB0007941 | 0.76036 | 0.82514 |
| Putrescine | HMDB0001414 | 0.76419 | 0.82514 |
| PC 37:1 | HMDB0007952 | 0.77072 | 0.82711 |
| PC 37:2 | HMDB0007954 | 0.80372 | 0.8573 |
| Sphinganine | HMDB0000269 | 0.83288 | 0.88306 |
| PC 42:5 | HMDB0008257 | 0.84713 | 0.88846 |
| PC 35:5 | HMDB0007951 | 0.84808 | 0.88846 |
| Ribulose-5-phosphate | HMDB0000618 | 0.8859 | 0.9148 |
| Asparagine | HMDB0000168 | 0.88829 | 0.9148 |
| Alanine | HMDB0000161 | 0.88881 | 0.9148 |
| 6-phosphogluconate | HMDB0001316 | 0.8993 | 0.92021 |
| Fructose-1,6-bisphosphate | N/A | 0.95289 | 0.96942 |
| alpha ketoglutarate | HMDB0000208 | 0.95913 | 0.97016 |
| PC 41:6 | N/A | 0.96955 | 0.97509 |
| Histidine | HMDB0000177 | 0.99152 | 0.99152 |

**Supplementary Figure 1. Principal component analysis (PCA) score plot of serum metabolome data obtained for sepsis-induced ARDS patients and non-ARDS controls: 12 QC samples were included.**

**
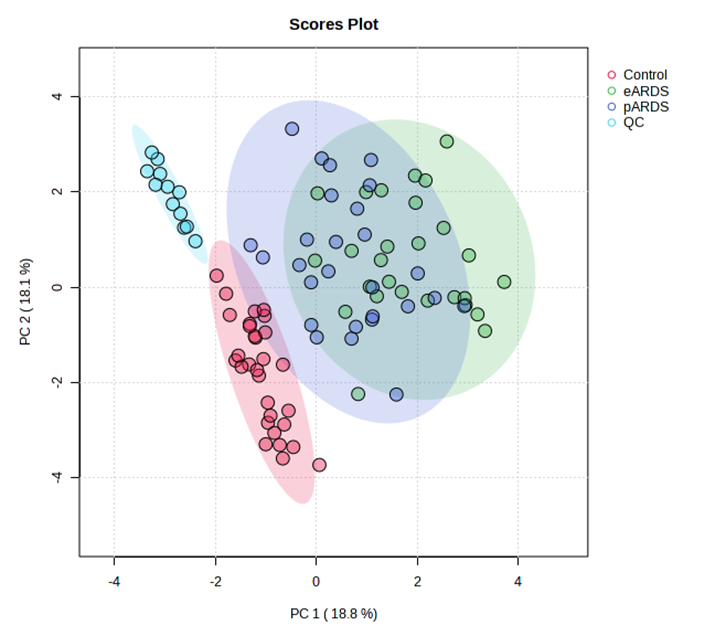
**

**Supplementary Figure 2. Statistical analysis of the data obtained for sepsis-induced ARDS patients and non-ARDS controls: PLS-DA cross-validation**


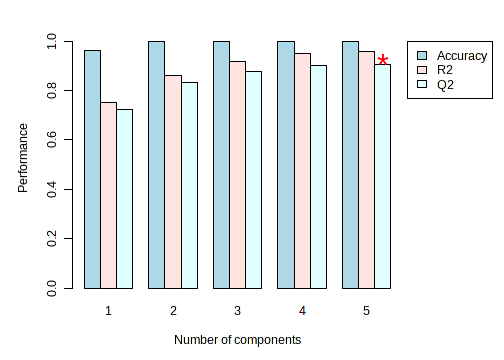

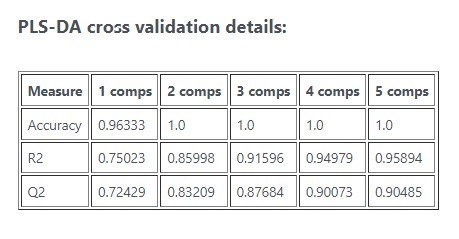


**Supplementary Figure 3. Pathway affected in sepsis-induced ARDS subphenotype**

**A. Direct ARDS compared to non-ARDS control**


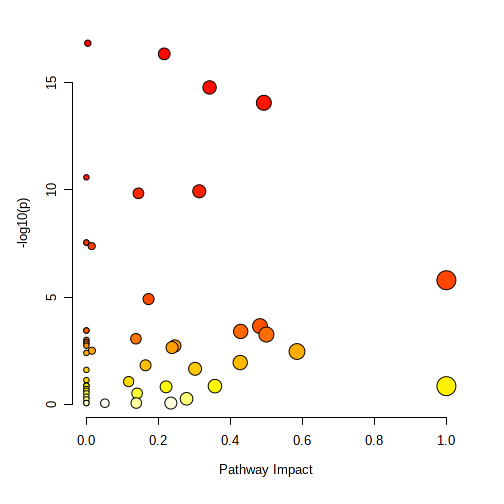


Glycolysis and gluconeogenesis

D-Glutamine and D-glutamate metabolism

Arginine biosynthesis

Fatty acid biosynthesis

Fatty acid degradation

Biosynthesis of unsaturated fatty acids

Ether lipid metabolism

Phenylalanine, tyrosine, and tryptophan biosynthesis

Linoleic acid metabolism

Arachidonic acid metabolism

Tryptophan metabolism

Sphingolipid metabolism

Glycerophospholipid metabolism

Glycosylphosphatidylinositol (GPI)-anchor biosynthesis

**B. Indirect ARDS compared to non-ARDS control**


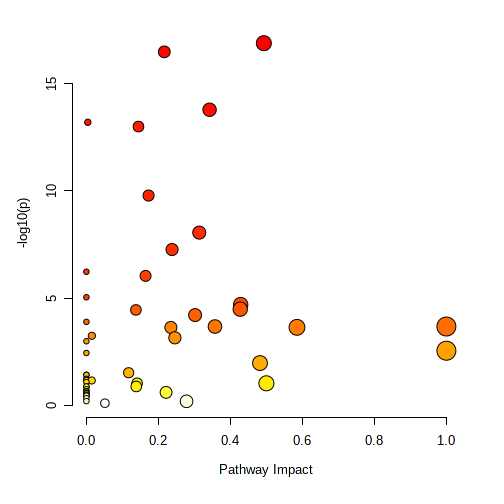


Phenylalanine, tyrosine, and tryptophan biosynthesis

Linoleic acid metabolism

Arachidonic acid metabolism

Tryptophan metabolism

Cysteine and methionine metabolism

Pyruvate metabolism

Tyrosine metabolism

Alanine, aspartate, and glutamate metabolism

Glycolysis and gluconeogenesis

Ether lipid metabolism

Glycosylphosphatidylinositol (GPI)-anchor biosynthesis

Glycerophospholipid metabolism

Sphingolipid metabolism

**C. Venn diagram for top 10 significant pathways for sepsis-induced direct and indirect ARDS compared to non-ARDS control**


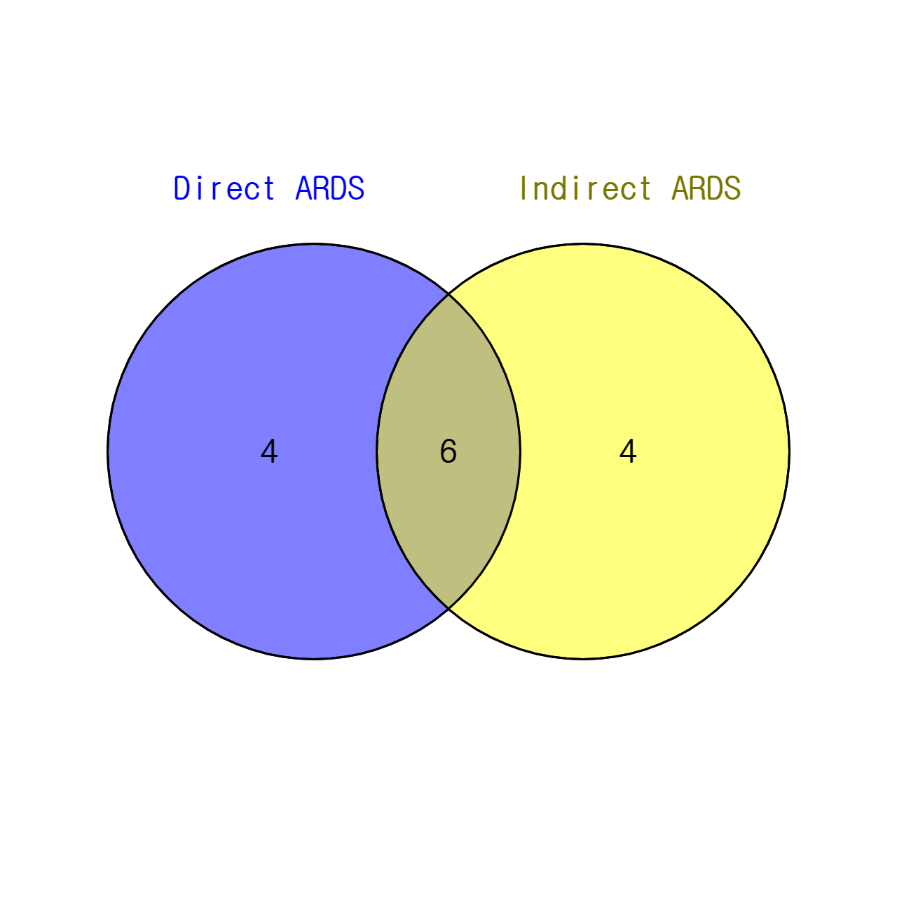


**4 exclusively in "Indirect ARDS"**

Glycolysis / Gluconeogenesis

Pyruvate metabolism

Cysteine and methionine metabolism

Tyrosine metabolism

**4 exclusively in "Direct ARDS"**

Biosynthesis of unsaturated fatty acids

Fatty acid elongation

Fatty acid degradation

Fatty acid biosynthesis

**6 common elements in "Direct ARDS" and "Indirect ARDS":**

Glycosylphosphatidylinositol (GPI)-anchor biosynthesis

Glycerophospholipid metabolism

Tryptophan metabolism

Sphingolipid metabolism

Arachidonic acid metabolism

Ether lipid metabolism
